# Non-Covalent Interactions between dUTP C5-Substituents and DNA Polymerase Decrease PCR Efficiency

**DOI:** 10.3390/ijms241713643

**Published:** 2023-09-04

**Authors:** Olga A. Zasedateleva, Sergey A. Surzhikov, Viktoriya E. Kuznetsova, Valeriy E. Shershov, Victor E. Barsky, Alexander S. Zasedatelev, Alexander V. Chudinov

**Affiliations:** Engelhardt Institute of Molecular Biology, Russian Academy of Sciences, 32 Vavilov Street, 119991 Moscow, Russia

**Keywords:** C5-modified dUTPs, A and B family DNA polymerases, PCR amplification, X-ray structure, molecular modeling, non-covalent interactions

## Abstract

The approach based on molecular modeling was developed to study dNTP derivatives characterized by new polymerase-specific properties. For this purpose, the relative efficiency of PCR amplification with modified dUTPs was studied using Taq, Tth, Pfu, Vent, Deep Vent, Vent (exo-), and Deep Vent (exo-) DNA polymerases. The efficiency of PCR amplification with modified dUTPs was compared with the results of molecular modeling using the known 3D structures of KlenTaq polymerase–DNA–dNTP complexes. The dUTPs were C5-modified with bulky functional groups (the Cy5 dye analogs) or lighter aromatic groups. Comparing the experimental data and the results of molecular modeling revealed the decrease in PCR efficiency in the presence of modified dUTPs with an increase in the number of non-covalent bonds between the substituents and the DNA polymerase (about 15% decrease per one extra non-covalent bond). Generalization of the revealed patterns to all the studied polymerases of the A and B families is discussed herein. The number of non-covalent bonds between the substituents and polymerase amino acid residues is proposed to be a potentially variable parameter for regulating enzyme activity.

## 1. Introduction

The regularities and mechanisms of the enzymatic incorporation of natural and modified nucleoside triphosphates by various DNA polymerases are intensively investigated [1,2,3]. The relevant studies allow for the creation of modified dNTPs to inhibit the polymerase’s enzymatic activity in pathogenic bacteria and viruses and not affect the polymerases of higher organisms [4,5,6,7]. Other aspects of this field include the creation of aptamers—enzymatically synthesized modified oligonucleotides capable of binding to target proteins with high specificity. This approach can be used to diagnose and treat infectious, cancer, and other diseases [8,9,10,11,12,13,14,15,16,17,18,19,20]. The key to this technology is the ability of various polymerases to synthesize DNA strands using nucleoside triphosphates with specific modifications in the structure of bases [21,22,23,24,25,26,27,28,29] and, in particular, to synthesize oligonucleotides with modifications to all bases [21,25,29].

3D-structural models of the enzymatic incorporation of natural and modified nucleotides are also investigated [1,30,31,32,33,34,35,36,37,38,39,40]. Based on X-ray diffraction studies, Steitz T.A. [34] and Rothwell P.J. and Waksman G. [35] found that, notwithstanding the significantly different amino acid composition, DNA polymerases of various organisms have a common shape resembling that of a half-open right hand and consisting of several subdomains—the so-called fingers, thumb, and palm [34,35]. These subdomains cover template fragment and synthesized DNA strand and form an active center where the complementary dNTP is localized. The dNTP is fixed in the necessary conformation by a set of non-covalent bonds with amino acid residues of the polymerase. The process is followed by the subsequent attachment of nucleotide to the growing DNA strand due to the formation of a phosphodiester bond and by the translocation of the substrate for a new round of incorporation [32,35].

Taq DNA polymerase is currently one of the most studied DNA polymerases. As shown by Waksman G. et al. [32], a large fragment of the Taq polymerase, KlenTaq, has open and closed forms, the so-called “relaxed” and “tight” structures, depending on the stage of nucleotide incorporation. The amino acid residues belonging to the active center of the Taq DNA polymerase (Tyr611, Ser612, Gln613, Ile614, Glu615, His639, the amino acid residues of O alpha helix—Arg659, Lys663, and Phe667 as well as Tyr671) form non-covalent interactions with potentially active chemical groups of the nucleotide being incorporated [32]. Arg660 residue also plays an essential role in incorporating a nucleotide stabilizing the complex’s structure due to forming a hydrogen bond with a phosphate group at the 3′ end of the primer DNA strand [32,33,36].

A significant study has been done by Marx A. et al. [36,37,38,39,40,41], who analyzed the structures of the “closed” DNA–KlenTaq polymerase complexes with modified dNTPs non-covalently bound to the active center of the enzyme. dNTPs bases were modified with substituents consisting of a linker and aliphatic or aromatic hydrocarbon functional groups. The enzymatic incorporation of these nucleotides occurs due to the presence of free spaces in the structure of the Taq polymerase adjacent to the active center. These free spaces, namely cavity A and cavity B, are located between the finger and the thumb or between the palm and the finger domains, respectively. More specifically, cavity A is “bordered by the Arg587 side chain and residues from the O-helix” while cavity B “runs parallel to the O-helix” [40]. When incorporating a modified nucleotide, the enzyme flexibly adapts its structure to various base modifications, redistributing interactions between amino acid residues and the 3′ end of the primer DNA strand. Polymerase forms additional non-covalent bonds with the substituent of the modified nucleotide depending on the structure of its linker and functional group. Arg587, Arg660, Ala661, Lys663, and Thr664 amino acid residues were shown to form non-covalent interactions with the substituents.

As noted by Marx A. et al. [36,38,39], the efficient enzymatic incorporation of modified nucleotides is facilitated by the chemical groups in the structure of the substituents capable of forming hydrogen or π–cationic bonds with amino acid residues of the DNA polymerase’s active center.

Hocek M. et al. [42] used a docking procedure to localize modified dNTPs at the previously known Bst polymerase–DNA complex structure. The π−cation interaction was shown to be between Arg629 of Bst polymerase and the phenyl group of dG^Ph^TP.

Our recent results [28] are somewhat consistent with the conclusions of Marx A. et al. [36,38,39]. Using the Taq polymerase as well as the Tth, Vent (exo-), and Deep Vent (exo-) polymerases, we have shown that the efficiency of PCR incorporation of deoxyuridines modified with aromatic hydrocarbon groups correlated with the hydrophilicity of these groups [28].

The authors of the above-cited articles used dNTPs with bases modified by 39–577 Da molecular weight substituents. Meanwhile, according to recent studies [43,44,45,46], enzymatic DNA synthesis may occur in the presence of dNTPs carrying bulkier substituents (molecular weights up to 692 Da). For example, Holliger P. et al. [43] showed the possibility of “colored” DNA synthesis by PCR with genetically modified DNA polymerase (mutant Pfu (exo-)–Pfu-E10) in the presence of dCTPs labeled with Cy3 or Cy5 fluorescent dyes.

Using an X-ray structural analysis for the complex of mutant polymerase Pfu (exo-)–Pfu-E10 with DNA and 3D modeling for Pfu-E10:DNA:Cy5-dCTP complex, Holliger, P. et al. [44] supposed that polymerase Pfu-E10 was able to incorporate Cy5-dCTP with high efficiency due to “the lack of major groove interactions between the polymerase and the DNA in the Pfu-E10:DNA complex”. The authors also suggested “that Cy5-dye modified bases could also be accommodated in the DNA duplex, with the dye molecules located in the major groove of the double stranded DNA”.

We found earlier [47] that the incorporation efficiency of dUMPs modified via linker with analogs of Cy3 and Cy5 cyanine dyes into the DNA chain by Taq polymerase significantly depends on the total charge of the fluorophores. In particular, incorporation efficiency is approximately ten times higher for dUMPs fluorescently labeled with electroneutral zwitterionic analogs of cyanine dyes than dUMPs labeled with negatively charged analogs of cyanine dyes.

In the present work, we tried to find the reasons of PCR efficiency variation for differently modified nucleotides using modified dUTPs as example. Seven DNA polymerases from families A and B (Taq, Tth, Pfu, Vent (exo-), Deep Vent (exo-), Vent, and Deep Vent) were used to study the efficiency of PCR amplification in the presence of dUTPs C5-modified with different substituents. Our experimental data on PCR efficiency for bulky substituents (536–694 Da) obtained in this study as well as those for lighter substituents (126–251 Da) demonstrated previously [28] were compared with the structural features of the substituents. For this purpose the structures of the substituents were virtually incorporated in the known X-ray structures of KlenTaq polymerase–DNA–(dUTP or ddTTP) 3D complexes [33,40,48] and optimized using covalent docking with help of the Discovery Studio program [49]. It was found that the increase in the quantity of non-covalent bonds between various dUTP C5-substituents and amino acid residues of KlenTaq polymerase resulted in a decrease in PCR efficiency. Therefore, it was proposed to consider the number of non-covalent bonds between the dNTP substituents and polymerase amino acid residues as a variable parameter useful in future design and synthesis of dNTP derivatives characterized by new polymerase-specific properties.

## 2. Results

### 2.1. PCR Efficiency Using Different DNA Polymerases and dUTPs C5-Modified with Bulky Substituents

#### 2.1.1. The Structures of C5-Attached dUTP Substituents and Electrophoretic Separation of PCR Products

**Figure 1:**
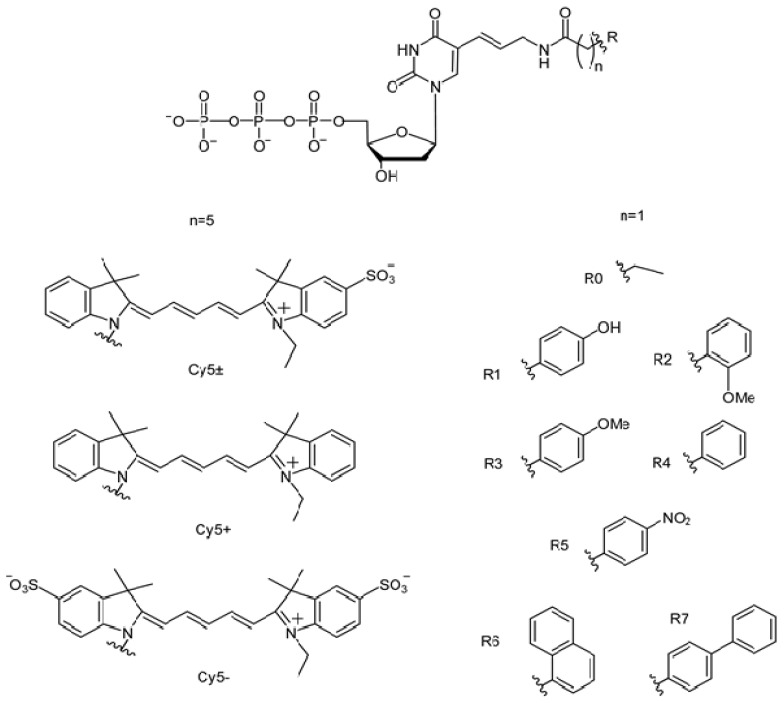
dUTPs modified at the C5 position of the pyrimidine ring with aromatic hydrocarbon groups via linker -CH=CH-CH_2_-NHCO-(CH_2_)_n_-. In the case of bulky substituents carrying Cy5 dye analogs, the linker part is longer (n = 5) while in the case of lighter substituents carrying R0–R7, the linker part is shorter (n = 1).

PCR efficiency was measured for seven A and B family DNA polymerases (Taq, Tth, Pfu, Vent (exo-), Deep Vent (exo-), Vent, and Deep Vent). In accordance with our previous data [50], PCR with Taq polymerase was most efficient in the presence of 5% of C5-modified dUTPs. Therefore, in the present study of PCR efficiency in the presence of dUTPs C5-modified with bulky substituents, 5% dTTP in the reaction mixtures was replaced by modified dUTPs. Figure 1 shows that modifications were carried out via a linker (-CH=CH-CH_2_-NHCO-(CH_2_)_5_-) at the C5 position of the uridine base of dUTPs using differently charged Cy5 dye analogs and denoted as dU(Cy5+)TP, dU(Cy5±)TP, and dU(Cy5–)TP. The synthesized structures of Cy5 dye analogs differ from each other by the number and localization of negatively charged SO_3_ groups.

**Figure 2:**
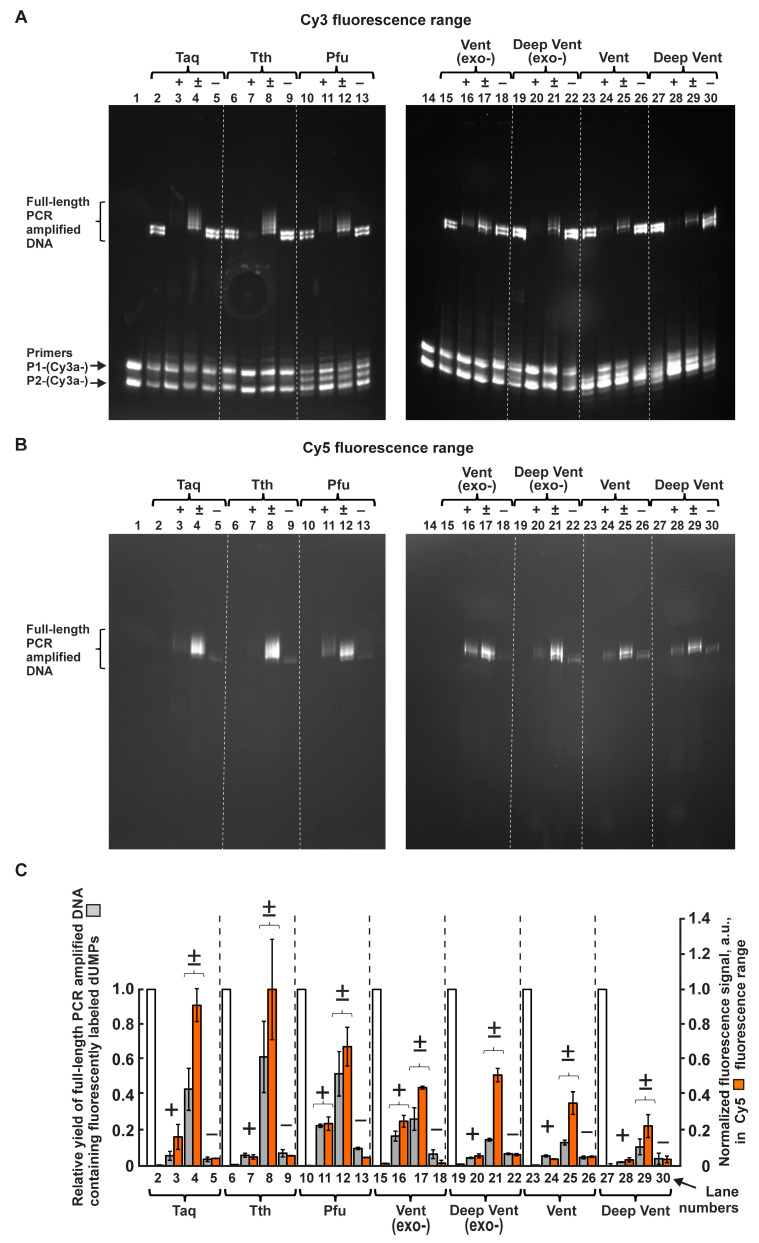
The efficiency of PCR amplification using seven A and B family DNA polymerases in the presence of dU(Cy5+)TP, dU(Cy5±)TP, or dU(Cy5–)TP in the reaction mixtures. PCR mixtures with modified dUTPs contained 10^−4^ M dATP, dTTP, dCTP, 0.95 × 10^−4^ M of dTTP, and 0.05 × 10^−4^ M of dU(Cy5+)TP, dU(Cy5±)TP, or dU(Cy5–)TP as correspondingly noted by the symbols (+, ± or –) in the plots. (**A**) Electrophoretic separations of PCR products are shown in the fluorescence range of the Cy3 dye (the labeling dye for both primers). Lanes 1 and 14 are the PCR mixtures without the polymerases. Lanes 2, 6, 10, 15, 19, 23, and 27 are the PCR mixtures containing dNTPs and Taq, Tth, Pfu, Vent (exo-), Deep Vent (exo-), Vent, or Deep Vent polymerases, respectively. Lanes 3, 7, 11, 16, 20, 24, and 28 are the PCR mixtures containing, respectively, the same polymerases and dATP, dCTP, dGTP, and 95% dTTP and 5% dU(Cy5+)TP. Lanes 4, 8, 12, 17, 21, 25, and 29 are the PCR mixtures containing, respectively, the same polymerases and dNTPs as well as 5% dU(Cy5±)TP. Lanes 5, 9, 13, 18, 22, 26, and 30 show the PCR mixtures containing, respectively, the same polymerases and dNTPs as well as 5% dU(Cy5–)TP. (**B**) Electrophoretic separations of PCR products are shown in the fluorescence range of the Cy5 dye (the bulky substituents of dUTPs contained its derivatives (Cy5+, Cy5±, or Cy5–)). (**C**) Histogram of the relative amounts of the full-size PCR-amplified DNA fragments. Grey columns represent the relative PCR efficiency obtained by normalizing the fluorescence intensities in the Cy3 dye fluorescence range of the DNA products containing incorporated modified dUMPs to the fluorescence intensities of the products obtained in the presence of only natural dNTPs. Orange columns show the fluorescence intensities of PCR products normalized to the maximum value in the Cy5 fluorescence range (for Tth polymerase). White columns reflect the relative fluorescent intensity of amplified DNA with only natural dNTPs in mixtures in the Cy3 fluorescence range.

Figure 2A,B show the electrophoregram of PCR products in polyacrylamide gel. The amount of PCR products was estimated after amplification in the presence of a 68-nucleotide template and two primers 18 and 17 nucleotides long, labeled at the 5′ end with Cy3 dye.

Figure 2A (left and right) and Figure 2B (left and right) show the images of gels in the fluorescence ranges specifically for Cy3 and Cy5 dye, respectively.

The experiments shown in A and B were performed twice. The average values from the two experiments are listed in Table S1 and presented in C. The bars indicate the absolute deviations.

The natural reaction products are visible in lanes 2, 6, 10, 15, 19, 23, and 27 (Figure 2A). The reaction products amplified in the presence of C5-modified dUTPs are in lanes 3–5 (for Taq polymerase), 7–9 (for Tth polymerase), 11–13 (for Pfu polymerase), 16–18 (for Vent (exo-)), 20–22 (for Deep Vent (exo-)), 24–26 (for Vent polymerase), and 28–30 (for Deep Vent polymerase).

Unspent primers are in the lower half of the gel images in Figure 2A. Polymerases with exonuclease activity, Pfu, Vent, and Deep Vent, cut off one nucleotide at the 3′ end of the primers, and thus, the primers have a couple of lighter fragments (lanes 10–13, 23–30).

#### 2.1.2. Inhibiting Effect of Modified dUTPs in the Reaction Mixture on the Yield of PCR Product Consisting Only of Natural Nucleotides

As can be seen in Figure 2, even low concentration (5 × 10^−6^ M) of dU(Cy5+)TP or dU(Cy5±)TP in the PCR mixture leads to significant synthesis inhibition of PCR products consisting of only natural nucleotides (lanes 3, 4, 7, 8, 11, 12, 16, 17, 20, 21, 24, 25, 28, 29). However, similar inhibition does not occur when the reaction mixture contains dU(Cy5–)TP at the same concentration (lanes 5, 9, 13, 18, 22, 26, 30). See the corresponding paragraph “Part A: Inhibiting effect of modified dUTPs in the reaction mixture on the yield of PCR product consisting only of natural nucleotides” in the supplementary data for details.

#### 2.1.3. Ratings of Seven A and B Family DNA Polymerases in Ability to Use Modified dUTPs in PCR

As seen in Figure 2A, for each polymerase, the PCR amplification in the mixture with 5% dU(Cy5±)TP leads to the appearance of heavier electrophoretic bands (lanes 4, 8, 12, 17, 21, 25, and 29) in comparison with the bands containing natural DNA product. 

Up to three such bands are visible in lanes 4, 8, 12, 17, 21, 25, and 29. These additional bands appear due to the weighting of the synthesized DNA strands when they contain, respectively, from 1 to 3 nucleotides that are C5-modified with a bulky substituent carrying Cy5± (see also Figure 1). These additional bands can also be observed for each DNA polymerase in the Cy5 fluorescence range (Figure 2B), though we could not resolve them in that range.

The values of relative fluorescence intensities in the Cy3 fluorescence range, characterizing the yields of the full-size PCR products containing nucleotide-modified DNA fragments, are summarized in Table S1 and plotted for each of the DNA polymerases in the histogram in Figure 2C (grey columns). The histogram also shows the relative fluorescence intensities in the Cy5 fluorescence range for the full-size PCR products containing Cy5-modified nucleotides (Figure 2C, orange columns). 

The data presented in Figure 2C enables us to rate the ability of DNA polymerases of the A and B families to use the dUTPs C5-modified with bulky Cy5 dye analogs in PCR amplification. For dU(Cy5±)TP, the rating can be approximately estimated as follows:

Tth ≥ Pfu ≥ Taq > Vent(exo-) > Deep Vent (exo-) ≥ Vent ≥ Deep Vent.(1)

At the same time, the rating for dU(Cy5+)TP is:

Pfu ≥ Vent(exo-) > Taq = Tth = Vent ≥ Deep Vent (exo-) > Deep Vent.(2)

Notably, the ability of the tested DNA polymerases to use dU(Cy5–)TP in PCR amplification is negligible.

#### 2.1.4. General Rating of dUTPs C5-Modified with Cy5 Dye Analogs According to PCR Efficiency with DNA Polymerases of the A and B Families

**Figure 3:**
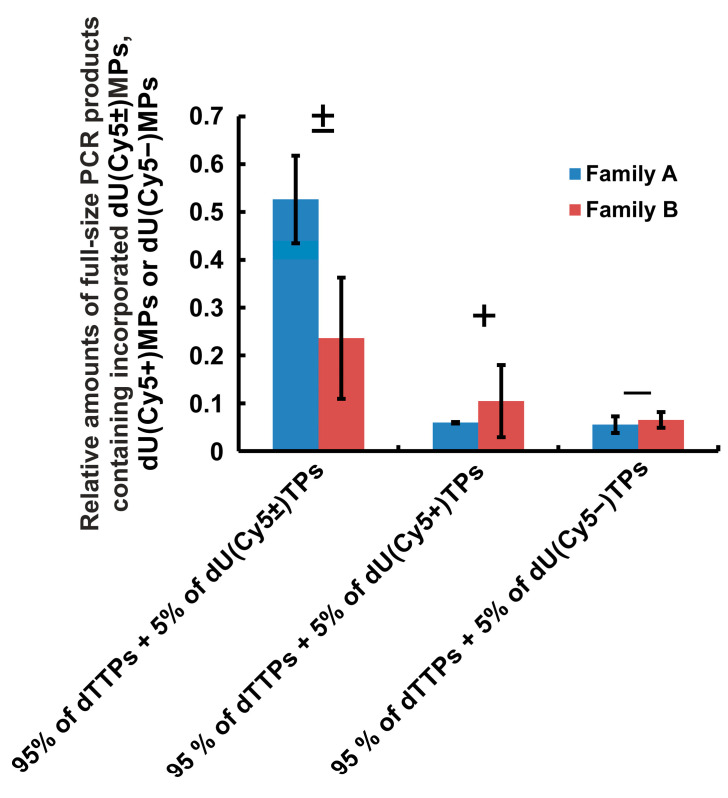
The relative amounts of full-size PCR products containing incorporated dU(Cy5±)MPs, dU(Cy5+)MPs, or dU(Cy5–)MPs averaged over the DNA polymerases of the A and B families compared to entirely natural PCR products. The relative amounts of the PCR products were taken from the experimental data presented in Figure 2 and Table S1. The bars indicate the mean absolute deviations.

Each polymerase synthesized the full-size PCR products containing incorporated dU(Cy5±)MPs less efficiently than entirely natural DNA products (Figure 2C and Figure 3) but more efficiently than the full-size DNA products modified by Cy5+ or Cy5–dye analogs.

Figure 3 shows that the efficiency of PCR amplification in the presence of modified dUTPs (as percentage from dTTP values) averaged independently for A family and B family DNA polymerases are (50 ± 10)% and (24 ± 13)% for dU(Cy5±)TP, (6 ± 1)% and (11 ± 8)% for dU(Cy5+)TP, and (6 ± 2)% and (7 ± 2)% for dU(Cy5–)TP, respectively. Thus, according to the efficiency of PCR amplification, the dNTPs are ranked in the following order:

dTTP > dU(Cy5±)TP > dU(Cy5+)TP ≥ dU(Cy5–)TP.(3)

This rating does not depend on whether the polymerase has 3′-5′ exonuclease activity, as in the case of Pfu, Vent, and Deep Vent polymerases, or does not have this activity, as in the case of Taq and Tth as well as in the cases when the polymerase is genetically modified so that the 3′-5′ exonuclease function is turned off, as in the case of Vent(exo-) and Deep Vent (exo-) polymerases.

### 2.2. Structural Factors Reducing the PCR Efficiency When dUTPs C5-Modified by Various Low Molecular Weight Substituents Are Used

The molecular modeling of possible conformations of the substituents at the C5 position of dUTPs localized in the active center of the KlenTaq polymerase was performed using the covalent docking procedure of Discovery Studio program [49] (a free version of the program was kindly provided by Dassault Systèmes software corporation). The substituents were virtually attached at the C5 position of pyrimidine rings of the known structures of the KlenTaq polymerase–DNA–(dUTP or ddTTP) “closed” triple complexes first determined by Waksman G. et al. [33] and later by Marx A. et al. [40] by X-ray structural analysis and published in the protein database [48] (PDB IDs: 1QTM and 5E41).

Before analyzing bulky aromatic hydrocarbon substituents (536–694 Da) attached at the C5 position of the pyrimidine ring of dUTP (characterized in Section 2.1), we analyzed the dUTPs modified using comparatively smaller and simpler substituents. For this purpose, the published experimental data [28] on the efficiency of PCR amplification using Taq, Tth, Vent (exo-), and Deep Vent (exo-) polymerases when replacing dTTP with dUTPs modified with small low molecular weight substituents (126–251 Da) containing R0–R7 functional groups (see Figure 1) were used.

Accordingly, the following low molecular weight substituents were virtually constructed (see Figure 1):

1. Small (126–251 Da) substituents consisting of -CH=CH-CH_2_-NHCO-CH_2_- linker and R0–R7 functional groups;
2. Bulky (536–694 Da) substituents consisting of -CH=CH-CH_2_-NHCO-(CH_2_)_5_- linker and the analogs of Cy5 dye.

#### 2.2.1. Non-Covalent Interactions between KlenTaq Polymerase and the Substituents Containing Small Functional Groups R0–R7

3D structures of the linker (-CH=CH-CH_2_-NHCO-CH_2_-) and the attached functional groups (R0–R7) were optimized by minimizing the relative energies of their conformations and interactions with surrounding chemical groups of polymerase and DNA. Meanwhile the remaining parts of the KlenTaq polymerase–DNA–(modified dUTP) complex maintain rigid conformation.

**Figure 4:**
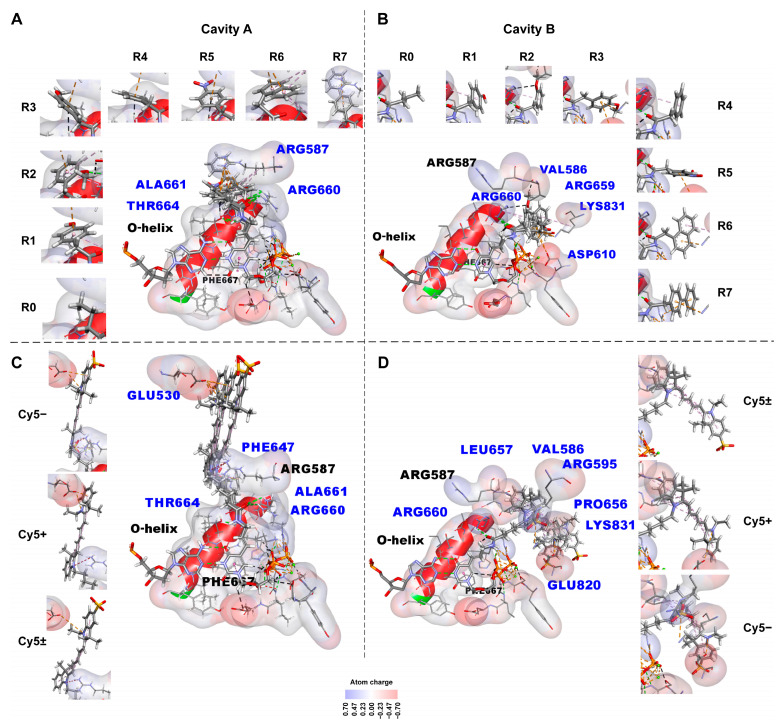
Fragments of three-dimensional structures of the KlenTaq polymerase–DNA–(modified dUTP or ddTTP) complexes (based on X-ray structures, PDB IDs 5E41, and 1QTM [33,40,48]). Computer-simulated conformations and localization of the low molecular weight substituents attached at the C5 position of the pyrimidine rings of the modified nucleotides incorporated in the active center of the enzyme are illustrated (.dsv files with these 3D structures are located in the supplementary data). (**A**,**B**)—the small substituents (126–251 Da) containing the functional groups R0–R7 attached using the -CH=CH-CH_2_-NHCO-CH_2_- linker. (**C**,**D**)—the bulky substituents (536–694 Da) containing the analogs of Cy5 dye (zwitterionic neutrally charged Cy5±, positively charged Cy5+, or negatively charged Cy5–) attached using the -CH=CH-CH_2_-NHCO-(CH_2_)_5_- linker. (**A**,**C**)—computer-simulated superpositions of the substituents are extended through cavity A of the 5E41 structure [48] of the enzyme (crossing over the O-helix). (**B**,**D**)—computer-simulated superpositions of the substituents are extended through cavity B of the 1QTM structure [48] of the enzyme (partially parallel to the O–helix). The interactions between the functional groups of each substituent with the neighboring amino acid residues are also shown in eight (**A**,**B**) or three (**C**,**D**) separate images detailing the corresponding superimposed structures.

The optimized conformations and the localization of small substituents of the modified dUTPs within the fragments of KlenTaq polymerase–DNA–(modified dUTP) 3D complexes are shown in Figure 4A,B. The substituents of the modified dUTPs can be oriented in two directions—via cavity A or cavity B, which are adjacent to the space of the active center of the KlenTaq polymerase [40]. We obtained that in the case of the 5E41 structure, the substituents extend through cavity A (Figure 4A), and in the case of the 1QTM structure, the substituents extend through cavity B (Figure 4B). 

Figure 4A,B also show various types of non-covalent bonds, namely:

hydrogen,
carbon–hydrogen,
π–donor hydrogen,
π–cation,
π–anion, and
π–alkyl bonds between the chemical groups of the substituents and the chemical groups of the amino acid residues of the KlenTaq polymerase obtained using the Discovery Studio program. These interactions are listed in Table S2. Table S2 also presents the mean number of non-covalent interactions between each substituent, and KlenTaq polymerase averaged over two types of localization (via cavity A and cavity B).

The amino acid residues of the KlenTaq polymerase forming non-covalent bonds with the substituents are indicated by blue characters in Figure 4A–D. The charge distribution over the surfaces bounding the van der Waals radii of the atoms is shown for amino acid residues of the active center of the KlenTaq polymerase using the standard scale of colors (plus—blue, minus—red).

The non-covalent bonds are shown using the Discovery Studio program [49] as follows:

- Hydrogen bonds by green dotted lines;
- Carbon and π–donor hydrogen bonds by black dotted lines;
- Electrostatic interactions, including π–cationic and π–anionic, by orange dotted lines;
- Hydrophobic π–alkyl interactions by purple dotted lines;
- Electrostatic attractive charge bonds by orange dotted lines;
- π–lone pair bond by green dotted line;
- π–sulfur bond by yellow dotted line;
- Hydrophobic π–π stacking bond is denoted by a pink dotted line, alkyl bonds by purple dotted lines, and the π–sigma bond by a dark purple dotted line. The bonds can be seen more in the Discovery Studio program [49] using dsv files with these 3D structures in the supplementary data. An unfavorable positive–positive interaction between the nitrogen atom close to the linker indolyl ring of the substituent and the nitrogen atom of Arg587 is marked by a red dotted line in C.

#### 2.2.2. Non-Covalent Interactions between KlenTaq Polymerase and Bulky Substituents

Figure 4C,D show sterically resolved and optimized conformations of the bulky substituents and their localization in KlenTaq polymerase–DNA–(modified dUTP) 3D complexes after energy minimization via covalent docking procedure. The orientation of the bulky aromatic substituents carrying Cy5 dye analogs is the same as for the small low molecular weight substituents shown in Figure 4A,B. Similar to the small aromatic substituents, the bulky aromatic substituents extend via cavity A for the 5E41 structure (Figure 4C) or via cavity B for the 1QTM structure (Figure 4D).

Figure 4C,D and Figure S1–S3 show various types of non-covalent bonds formed between the bulky aromatic substituents and the amino acid residues of the KlenTaq polymerase. These non-covalent bonds are:

hydrogen,
electrostatic (attractive charge bonds, π–anion bonds),
π–lone pair bond,
π–sulfur bond, and
hydrophobic bonds (π–π-stacked, π–sigma, alkyl, and π–alkyl bonds), which are also listed in Table S3. Table S3 also presents the mean numbers of non-covalent bonds between the chemical groups of the substituent, and the chemical groups of the KlenTaq polymerase amino acid residues averaged over two types of localization (via cavity A and cavity B) for each substituent.

#### 2.2.3. Negative Correlation between PCR Efficiency and the Number of Non-Covalent Bonds between the dUTP C5-Substituents and DNA Polymerase 

##### Correlations for dUTPs C5-Modified with R0–R7 Functional Groups 

**Figure 5:**
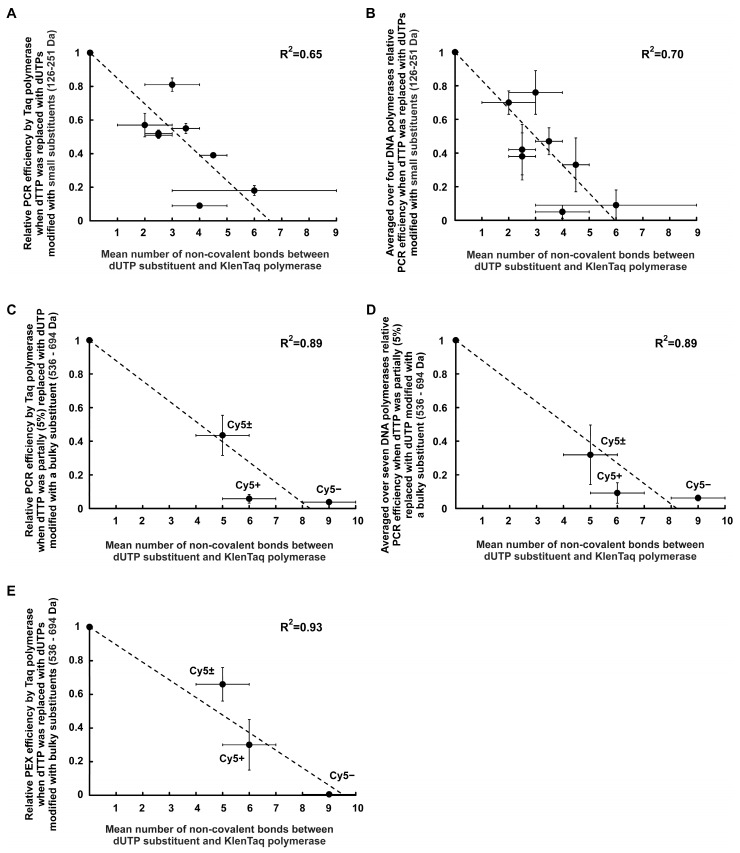
Correlations between the relative PCR or PEX efficiency in the presence of C5-modified dUTPs and the number of non-covalent bonds formed by the dUTP substituents with the KlenTaq polymerase amino acid residues. (**A**) Y-axis shows the relative PCR efficiency of the Taq polymerase when all the dTTP molecules were replaced with dUTPs modified with small substituents (126–251 Da) carrying functional groups R0–R7; (**B**) Y-axis shows the relative PCR efficiency averaged over four polymerases (Taq, Tth, Vent (exo-), and Deep Vent (exo-)) when all the dTTP molecules were replaced with dUTPs C5-modified with small substituents ((126–251 Da) carrying functional groups R0–R7; (**C**) Y-axis shows the relative PCR efficiency of the Taq polymerase when 5% dTTP molecules were replaced with the dUTPs modified with bulky aromatic substituents (536–694 Da) carrying Cy5 dye analogs of various total charges, i.e., Cy5±, Cy5+, or Cy5−; (**D**) Y-axis shows the relative PCR efficiency averaged over seven polymerases (Taq, Tth, Pfu, Vent, Deep Vent, Vent (exo-), and Deep Vent (exo-)) when 5% dTTP molecules were replaced with dUTPs modified by bulky aromatic substituents; and (**E**) Y-axis shows the relative PEX efficiency using the Taq polymerase when all the dTTP molecules were replaced with the dUTPs modified by bulky aromatic substituents. The bars indicate mean absolute deviations. The dotted straight lines show the linear approximations for the corresponding values.

Figure 5A shows the plot of experimentally estimated values of the relative PCR efficiency (for Taq polymerase in the presence of dUTPs C5-modified with R0–R7 functional groups) depending on the mean number of non-covalent bonds between the substituents and the polymerase. These mean numbers were calculated for each substituent by averaging the numbers of substituent–polymerase bonds estimated by docking for two possible localizations of the substituent in the enzyme (through the A cavity or the B cavity). This plot demonstrates a negative correlation (characterized by a linear decrease of about 15% per one non-covalent bond, R^2^ = 0.65) between the relative efficiency of PCR amplification (in the presence of modified dUTP) and the mean number of non-covalent bonds (formed by the dUTP C5-substituent with the KlenTaq polymerase amino acid residues). Notably, a similar negative correlation (R^2^ = 0.70, see Figure 5B) takes place if the values of the relative PCR efficiency averaged over the polymerases studied in [28] (Taq, Tth, Vent (exo-), and Deep Vent (exo-)) are plotted on the y-axis (instead of the relative PCR efficiency values for the Taq polymerase only). 

X-axis in all the plots of Figure 5 shows the mean number of non-covalent bonds between dUTP substituents and KlenTaq polymerase. The numbers of non-covalent bonds were obtained by molecular modeling based on the known X-ray structures [33,40,48] and averaged on localizations within 5E41 and 1QTM structures (see also Tables S2 and S3 and Figure 4 and Figure S1–S3). PCR efficiency is plotted as a percentage with that in the presence of only natural dNTPs. 

##### Correlations for dUTPs C5-Modified with the Bulky Substituents

Figure 5C shows the experimentally obtained values of the relative PCR efficiency obtained for the Taq polymerase in the presence of dUTPs C5-modified by the bulky substituents (containing Cy5+, Cy5±, or Cy5−; see Figure 1 and Table S3) depending on the mean number of non-covalent bonds between the substituents and the polymerase. As for small low molecular weight aromatic substituents (see Figure 5A), the plot in Figure 5C demonstrates a linear decrease of the relative PCR efficiency for about 12% per one non-covalent bond, R^2^ = 0.89 for the case of bulky substituents.

Thus, dUTPs C5-modified with bulky substituents can be characterized as follows:

For dU(Cy5±)TP:
relative PCR efficiency—0.44 ± 0.12; mean number of non-covalent bonds—(5 ± 1);
For dU(Cy5+)TP:
relative PCR efficiency—0.06 ± 0.03; mean number of non-covalent bonds—(6 ± 1);
For dU(Cy5–)TP:
relative PCR efficiency—0.04 ± 0.01; mean number of non-covalent bonds—(9 ± 1).

The plots in Figure 5A,C demonstrate that PCR amplification efficiency via Taq polymerase in the presence of modified dUTPs is decreased when increasing the number of non-covalent interactions formed between the dUTP C5-substituent and the polymerase. 

Figure 5D shows PCR efficiency values averaged over all the polymerases used in our work (Taq, Tth, Pfu, Vent, Deep Vent, Vent (exo-), and Deep Vent (exo-)) and plotted on the y-axis. Similar for small substituents (Figure 5B), the negative correlation (R^2^ = 0.89) was found for the PCR efficiency values averaged over all the tested polymerases for bulky substituents (Figure 5D).

Summing the obtained results, one can conclude that the number of non-covalent bonds between the dUTP C5-substituent and the amino acid residues of polymerase characterizes the ability of polymerase to incorporate modified nucleotide. The established pattern can be used to design and synthesize dNTP derivatives characterized by new polymerase-specific properties.

#### 2.2.4. Negative Correlation between PEX Efficiency and the Number of Non-Covalent Bonds Formed by dUTP C5-Substituents with the Taq Polymerase

Figure 5E shows the relative efficiency of PEX by Taq polymerase in the presence of dUTPs C5-modified with bulky aromatic substituents on the number of non-covalent bonds between the substituents and the amino acid residues of the KlenTaq polymerase. The relative efficiencies of PEX were taken from our recent paper [47]. In that paper, PEX experiments were carried out with dU(Cy5+)TP, dU(Cy5±)TP, and dU(Cy5–)TP and a similar DNA template.

The dependences of the relative PCR (Figure 5C) and PEX (Figure 5E) efficiencies on the docking-obtained quantity of non-covalent interactions between the dUTP C5-substituent and Taq polymerase looks quite similar. Thus, both PCR and PEX approaches, combined with molecular modeling, are applicable for analyzing non-covalent interactions between the dNTP substituent and the polymerase affecting the latter’s functioning.

#### 2.2.5. The Similarity of the Local Environments of the dUTP C5-Substituents Localized in the Active Center of Different Polymerases

Based on the similarity of the plots shown in Figure 5A,B and the plots shown in Figure 5C,D, one can propose that the chemical groups of amino acids facing the spaces of active centers of DNA polymerases as well as those facing the adjacent regions (possibly forming cavities similar to the A and B KlenTaq polymerase cavities) form similar local environments. These local environments are similar not only for dUTP (that is well known), but also for its substituents attached at the C5 position of the pyrimidine ring.

It can be assumed that modified nucleotides incorporated under different polymerases is localized in a similar environment of chemical groups despite the varieties in the amino acid chains forming the active centers of different polymerases. For details, see the corresponding paragraph “Part E: The similarity of local environments in the active centers of various polymerases to the dUTP substituents attached at the C5 position of the pyrimidine ring” in the supplementary data.

#### 2.2.6. Analysis of the Linker Parts of the dUTP C5-Substituents in Non-Covalent Interactions with Taq Polymerase

Analysis of the -CH=CH-CH_2_-NHCO-CH_2_- and -CH=CH-CH_2_-NHCO-(CH_2_)_5_- linker parts of the modeled substituents show that mainly the NH group and oxygen atom of the “inverted” peptide group, NHCO, of both linkers are involved in the formation of non-covalent (hydrogen) bonds with the atoms of the polymerase amino acid residues (Figure 4). The NH group or oxygen atom (or both) are involved in interactions. Meanwhile, for the substituentcarrying R6 functional group, a single carbon–hydrogen bond is formed between the carbon atom (next to the nitrogen atom of the NHCO group) of the linker and the oxygen atom of the Arg660 CONH group.

#### 2.2.7. Analysis of the Functional Groups R0–R7 of the dUTP C5-Substituents in Non-Covalent Interactions with Taq Polymerase

Structural models presented in Figure 4A,B show that the aromatic rings of R1–R7 functional groups form non-covalent (π–donor hydrogen, electrostatic, and/or hydrophobic) interactions with the amino acid residues of the KlenTaq polymerase. Meanwhile, the chemical groups attached to the aromatic rings (see functional groups R1–R7 in Figure 1) do not participate in the interactions with the polymerase chemical groups.

The exception is the 2-OMe group in R2. The oxygen atom of the 2-OMe group in R2 is involved in the hydrogen bond with the nitrogen atom of Arg660 in the case of the 5E41 structure (Figure 4A) and the carbon–hydrogen bond with the carbon atom of Arg660 in the case of the 1QTM structure (Figure 4B). The carbon atom of the 2-OMe group in R2 is also involved in the carbon–hydrogen bond with the oxygen atom of Val586 in the case of the 1QTM structure.

The formation of the non-covalent bonds between 2-OMe group of R2 and the polymerase can be the reason of 1.5–2 times lower PCR efficiency of Taq, Vent (exo-), and Deep Vent (exo-) polymerases in the presence of R2-modified dUTPs as compared with dUTPs conjugated with a 4-methoxyphenyl residue (R3) [28] whose 4-OMe group does not form non-covalent interactions.

#### 2.2.8. Analysis of Bulky dUTP C5-Substituents in Non-Covalent Interactions with Taq Polymerase

Figure S3 shows that the negatively charged bulky substituent of dU(Cy5–)TP forms more non-covalent bonds with the polymerase, (9 ± 1 bonds), than neutral (5 ± 1 bonds) or positively charged (6 ± 1 bonds) bulky substituents (Cy5 dye analogs). The negatively charged substituent differs from neutral or positively charged ones by the additional SO_3_ group attached to the chromophore indolyl ring closest to the linker. This SO_3_ group forms two extra non-covalent bonds with the amino acid residues of the Taq polymerase in both structures: 5E41 and 1QTM. In the case of the 5E41 structure, the aromatic ring of Phe647 forms a π–sulfur bond with the sulfur atom of the SO_3_ group and a π–lone pair bond with the oxygen atom of the SO_3_ group (see Figure S3A). In the case of the 1QTM structure, the nitrogen atoms of Arg595 and Lys831 form two electrostatic attractive charge bonds with the sulfur atom of the SO_3_ group (see Figure S3B). Due to these extra bonds and the other non-covalent bonds (6 in the case of 5E41 structure and 8 in the case of 1QTM structure), dU(Cy5–)MP probably has a lower efficiency of inclusion during PCR or PEX.

Notably, indolyl rings of the substituents, which are closest to the linker in both dU(Cy5+)TP and dU(Cy5±)TP, do not contain SO_3_ groups in the contrary to dU(Cy5–)TP. Respectively, neutral and positively charged substituents form a lower number of non-covalent bonds, (6 ± 1 bonds) and (5 ± 1 bonds) correspondingly (see Figures S1 and S2 and Table S3).

The considered numbers of non-covalent bonds are the extra ones compared to their absence in dUTP. These extra bonds and the corresponding energies of interactions should be overcome to release the polymerase molecule for the movement along the DNA primer/template just after the formation of the phosphodiester bond.

#### 2.2.9. Analysis of Amino acid Residues Capable of Forming Non-Covalent Bonds with the dUTP-Attached Substituents

**Figure 6:**
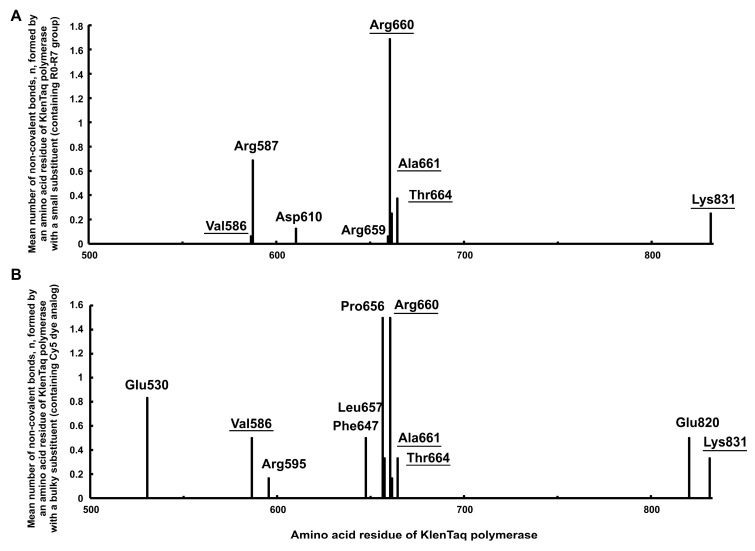
The mean number of non-covalent bonds, n, between the KlenTaq polymerase amino acid residues and the substituents (attached at the C5 position of the pyrimidine base of the dUTPs) containing functional groups R0–R7 (**A**) or Cy5 dye analogs (**B**).

Figure 6A shows the result obtained for small low molecular weight aromatic substituents (126–251 Da). Eight amino acid residues are detected with the help of molecular modeling (Val586, Arg587, Asp610, Arg659, Arg660, Ala661, Thr664, and Lys831) as capable of forming non-covalent bonds with the dUTP-attached substituents. Five of them (Arg587, Arg659, Arg660, Ala661, and Thr664) are included in the active center of the enzyme, while other three (Val586, Asp610, and Lys831) face cavity B adjacent to the active center of the KlenTaq polymerase. The amino acid residues Arg587, Arg660, Ala661, and Thr664 found by this modeling were first observed by Marx A. et al. ([36,37,38,39,40,41]; see Introduction), who found one more amino acid residue, namely Lys663.

The X-axis in Figure 6 shows the sequence number of the amino acid residue in the Taq polymerase chain. The Y-axis shows the mean number of non-covalent bonds per one structural solution. The data were obtained using molecular modeling and covalent docking for the substituents of the modified dUTPs localized in the structures 1QTM and 5E41. The amino acid residues forming non-covalent interactions with both small and bulky substituents are underlined and correspondingly shown in the histograms A and B. The n values were calculated as described in the Materials and Methods, Section 4.6 and detailed in “Part F: Table S4” in the supplementary data.

Similarly, Figure 6B shows that the amino acid residues participating in the interactions with bulky aromatic hydrocarbon substituents are Glu530, Val586, Arg595, Phe647, Pro656, Leu657, Arg660, Ala661, Thr664, Glu820, and Lys831. Among these residues, Glu530, Val586, Arg595, Phe647, Pro656, Leu657, Glu820, and Lys831 extend beyond the active center of the polymerase. The amino acid residues Glu530 and Phe647 face cavity A while the amino acid residues Val586, Arg595, Pro656, Leu657, Glu820, and Lys831 face cavity B.

Totally 14 KlenTaq polymerase amino acid residues (Glu530, Val586, Arg587, Arg595, Asp610, Phe647, Pro656, Leu657, Arg659, Arg660, Ala661, Thr664, Glu820, and Lys831) are detected with the help of molecular modeling and docking and identified as capable of forming non-covalent bonds with the chemical groups of both light (126–251 Da) or bulky (536–694 Da) low molecular weight aromatic substituents. Among these amino acid residues, five, Val586, Arg660, Ala661, Thr664, and Lys831 (underlined in Figure 6A,B), can form non-covalent interactions with both small and bulky substituents (see also Materials and Methods, Section 4.6 and “Part F: Table S4” in the supplementary data).

## 3. Discussion

The analysis of the structures of DNA polymerase–DNA–(modified dNTP) 3D complexes aims to understand the mechanisms of the enzymatic incorporation of modified nucleotides into the DNA chain. This is essential to find optimal structures of functional dNTP substituents necessary to meet many biotechnological and medical challenges [51]. Usually, this analysis is time consuming as it is rather empirical and based on direct experimental verification. The correlation between the experimental data and the molecular modeling results obtained in the present study might be applied to facilitate the search for specific substituent structures. The described structural–functional relationships would probably allow one to analyze the properties of modified dNTPs using preliminary computational methods. This approach aims to either increase the efficiency of polymerases or use modified dNTPs as their selective inhibitors. The regularities identified in this study are considered and discussed below.

### 3.1. Negative Correlation between the PCR Efficiency and the Number of Non-Covalent Bonds between the dUTP C5-Substituents and DNA Polymerase

A negative correlation between the number of non-covalent bonds formed by dUTP-attached substituents with the KlenTaq polymerase amino acid residues and the PCR amplification efficiency in the presence of the modified dUTPs was found by comparing the experimental data with the results of molecular modeling. Roughly, the PCR efficiency decrease is about 15%, with an increase per one non-covalent bond between the substituent and the polymerase. This correlation turns out to be true for all the studied polymerases.

Molecular modeling has revealed various types of non-covalent bonds responsible for the specificity of dNTP C5-substituent-polymerase interactions: hydrogen, carbon–hydrogen, π–donor hydrogen, electrostatic (attractive charge bonds, π–cation, π–anion bonds), π–lone pair bond, π–sulfur bond, and hydrophobic bonds (π–π-stacked, π–sigma, alkyl, and π–alkyl bonds). This set of interactions obtained in molecular modeling can be helpful in the choice of structural solutions for new polymerase-specific dNTP derivatives in the future.

Thus, the number of non-covalent interactions between the substituents and the polymerase amino acid residues can be a potentially variable parameter for regulating the enzyme activity. 

### 3.2. The Similarity of the Local Environments for dNTP Substituents Localized in the Active Centers of Different Polymerases

It was found that various polymerases of the A and B families (Taq, Tth, Pfu, Vent (exo-), Deep Vent (exo-), Vent, and Deep Vent) possess a reduced ability (but not too different in values) to incorporate the dUMPs modified with small [28] or bulky aromatic substituent into the growing DNA strand as compared with natural dTMP (see the conclusions in [28], Figure 2C and Figure 3, and Relationship (3)). This result demonstrates the similarity of all the tested DNA polymerases of the A and B families in the ability to use a substrate that mimics dTTP, the dUTP modified with a certain substituent. 

Our data suggest the similarity of the local environments of dNTP substituents in the active centers of various polymerases. For example, covalent docking used in the present study (see Table S2) show the formation of π–cation bonds between phenyl rings of functional groups R1–R7 of dU*TP substituents and positively charged amino acid residues Arg587 (in the case of the 5E41 structure) or Lys831 and Arg659 (in the case of the 1QTM structure) of KlenTaq polymerase. Recently, Hocek M. et al. [42], also published the formation of a π–cation interaction between the phenyl ring of the dG*TP substituent and Arg629 of another enzyme—Bst polymerase—using docking. Thus, molecular modeling shows that the positively charged polymerase amino acid residues of at least two DNA polymerases form the π–cation bonds with phenyl rings of dU*TP, or dG*TP aromatic substituents. The existence of π–cation interactions between the phenyl ring of the dT*TP or dC*TP substituent and Arg587 and Lys663 of the KlenTaq polymerase was previously shown by Marx A. et al. in X-ray studies [38].

Moreover, as was published by Marx A. et al. [52], the same substituent of modified dATP (7- deaza-modified adenosine triphosphate) forms three similar non-covalent bonds for both the KOD DNA polymerase and KlenTaq DNA polymerase. In the case of the KOD polymerase, the substituent forms a hydrogen bond and a carbon–hydrogen bond with Lys487 and an alkyl bond with Ile488 (6Q4T [48,52]). In contrast, in the case of the KlenTaq polymerase, the same substituent forms two hydrogen bonds with Thr664 and an alkyl bond with Lys663 (6Q4U [48,52]).

Thus, based on the abovementioned results as well as on the similarity in the secondary structures of DNA polymerases [34,35], one can assume that, despite the varieties in the amino acid chains forming the active centers of different polymerases, the chemical groups of the amino acid residues facing the spaces of the active centers as well as those facing the adjacent regions (possibly forming cavities similar to cavities A and B of the KlenTaq polymerase) form similar local environments not only for dNTPs (that is well known), but also for their substituents.

### 3.3. The Role of Non-Covalent Interactions between Low Molecular Weight Substituents of the Modified dNTPs and the DNA Polymerase in Enzymatic Incorporation Efficiency

Marx A. et al. [36,38,39,40] performed X-ray crystallography for many structures. In the paper [38], he wrote that concerning the modified dNTPs, “hydrogen-bonding capability might improve their substrate properties” and “the introduction of an aromatic ring enables new interactions as π–cation interaction to positively charged amino acid side chains, such as arginine or lysine. This fact might explain the efficient processing of dT*TP and dC*TP”. 

Meanwhile, molecular modeling and comparing it with experimental data lead us to the opposite conclusion: the relative efficiency of PCR or PEX in the presence of modified dNTPs decreases with an increase in the number of non-covalent bonds between the substituent and polymerase. These non-covalent bonds may be hydrogen, carbon–hydrogen, π–donor hydrogen, electrostatic (attractive charge bonds, π–cation, π–anion bonds), π–lone pair bond, π–sulfur bonds, and hydrophobic bonds (π–π-stacked, π–sigma, alkyl, and π–alkyl bonds). The data presented in Figure 5A–E for the modified dUTPs may be an example.

#### 3.3.1. Incorporation Efficiency of dT^spin^MP and dT^dend^MP and X-ray Structural Data Obtained by Marx A. et al. Analyzed in the Discovery Studio Program

Our conclusion concerning the decrease of incorporation efficiency due to the number of non-covalent bonds between the dUTP substituent and polymerase is in agreement with the previous X-ray data and the results of competitive incorporation of modified dNTPs in the presence of their natural counterparts [36,37,38,39,40]. Kinetic experiments published by Marx A. et al. [36] showed that dT^spin^MP (where the small “spin” substituent had a mass of 163 Da) was incorporated by the KlenTaq polymerase with surprisingly less efficiency than dT^dend^MP (where the bulkier “dend” substituent had a mass of 577 Da). It would be interesting to understand why a substituent with a significantly larger mass (“dend”) inhibits the inclusion of the corresponding nucleotide to a lesser extent than a substituent with a smaller mass (“spin”).

In attempting to explain this apparent discrepancy, we analyzed X-ray structures obtained by Marx A. et al. [36], namely KlenTaq polymerase–DNA–(dT^spin^TP or dT^dend^TP) complexes using the Discovery Studio program [49]. We downloaded the corresponding structure files 3OJU.PDB and 3OJS.PDB from the Protein Data Bank [48] and compared the number of non-covalent bonds formed by “spin” and “dend” substituents with the amino acid residues of the KlenTaq polymerase. It was obtained that two non-covalent carbon–hydrogen bonds were formed by the small “spin” substituent (between the oxygen atom of the “spin” substituent and the carbon atoms of Arg587 and Arg660, respectively). In contrast, only one non-covalent hydrogen bond was formed by the bulky “dend” substituent (between the oxygen atom of the “dend” substituent and the nitrogen atom of Arg587). This fact can be considered in favor of the proposal that a lower number of dNTP substituent–polymerase non-covalent interactions facilitates the enzymatic incorporation of such modified dNMP. 

Meanwhile, using the Discovery Studio program, we could ascertain that the “dend” substituent additionally forms five non-covalent bonds with DNA (two hydrogen bonds with a previous nucleotide, dCMP; one π–lone pair bond; and two carbon–hydrogen bonds with the complimentary dAMP). We suppose that these substituent–DNA bonds improve incorporation efficiency at the first step of modified dNTP binding to the enzyme’s active center. However, at the same time, these DNA–substituent interactions do not decrease the incorporation efficiency since they do not need to be broken for the subsequent one-step translocation of the polymerase for attaching the next nucleotide.

#### 3.3.2. Competitive Incorporation of the Modified dNTPs and the X-ray Structural Data for KlenTaq Polymerase–DNA–(Modified dNTP) Complexes

To more widely test the usefulness of the developed approach and explain the results recently obtained in different works, we used the data from X-ray structural studies published in recent papers for six substituents in the corresponding twelve X-ray structures, 4DFM, 4DMJ, 4DFP, 4DF8 [37], 4ELT, 4ELU [38], 4DFK, 5SZT, 5E41, 4DF4 [40], 6Q4U, and 6Q4T [52], obtained by Marx A. et al. The structure files were downloaded from the Protein Data Bank [48].

**Figure 7:**
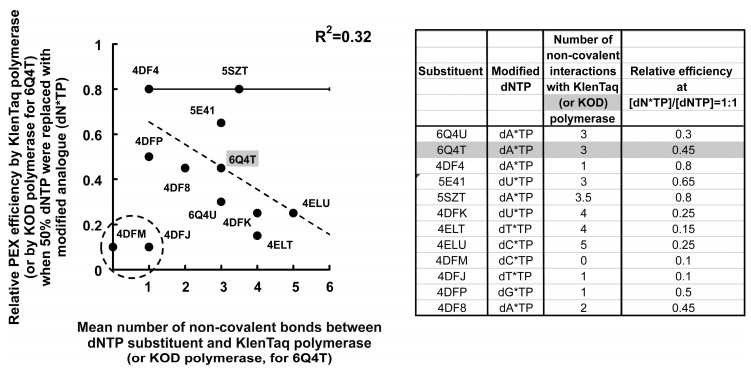
Correlation between the relative PEX efficiency and the number of non-covalent bonds that each of the dNTP substituents forms with amino acid residues of the KlenTaq polymerase [37,38,40,48,52] or KOD polymerase (for 6Q4T [48,52]). PEX efficiency was measured when 50% dNTP was replaced with dNTP modified with the following low molecular weight substituent (82–577 Da) (dN*TP): 4DF8, 4DFP, 4DFJ, 4DFM [37], 4ELU, 4ELT [38], 4DFK, 5SZT, 5E41, 4DF4 [40], 6Q4T, 6Q4U [52]. The ordinate axis shows the relative PEX efficiency when 50% dNTP was replaced with dN*TP using the KlenTaq polymerase [37,38,40,52] or KOD polymerase [52]. The abscissa axis shows the number of non-covalent bonds between dNTP substituents and the KlenTaq or KOD polymerase obtained from X-ray studies [37,38,40,48,52]. For the structure 5SZT, the number of non-covalent bonds is averaged over extensions through cavities A and B, and the bars indicate the mean absolute deviations. The dotted straight line shows linear approximations for the corresponding values. The table shows the plot data.

Figure 7 shows the relative PEX efficiency values estimated by Marx A. et al. [37,38,40,52] at dN*TP/dNTP = 1:1 (y-axis) plotted against the structure-determined numbers of non-covalent substituent–polymerase bonds (x-axis). Ten out of twelve points on the plot form a negative correlation while two points corresponding to the structures 4DFM and 4DFJ (circled) are outside. The resulting correlation is characterized by a linear decrease of about 10% per one non-covalent bond, roughly similar to the case of a decrease in the relative efficiency of PCR (15% per one non-covalent bond), as demonstrated in Figure 5A. The corresponding values of the parameters are included in the table near the plot in Figure 7.

Notably, each point of the plot was obtained in independent experiments using different nucleotides modified with different substituents. Therefore, one can conclude that non-covalent interactions between dNTP substituents and polymerases really decrease enzymatic incorporation efficiency.

Thus, it can be concluded that non-covalent interactions between low molecular weight substituents of the modified dNTPs and the DNA polymerase stabilize the localization of the modified dNTPs in the active center of the enzyme and increase the efficiency of the phosphodiester bond formation. However, the energy of non-covalent bonds should be overcome by their breaking for the subsequent one-step translocation of the polymerase to attach the next nucleotide.

To complete the entire cycle of enzymatic nucleotide incorporation, the energy loss due to the breaking of non-covalent bonds to continue the polymerase movement along the DNA template is more important than the energy gain of localization of the modified dNTP in the enzyme’s active center. 

### 3.4. Analysis of Amino Acid Residues of the KlenTaq Polymerase Capable of Forming Non-Covalent Bonds with the dUTP C5-Substituents

The molecular modeling using the Discovery Studio program [49] and X-ray structures 1QTM and 5E41 [33,40,48] revealed 14 KlenTaq polymerase amino acid residues (Glu530, Val586, Arg587, Arg595, Asp610, Phe647, Pro656, Leu657, Arg659, Arg660, Ala661, Thr664, Glu820, and Lys831) that can potentially form non-covalent bonds with the chemical groups of comparatively light (126–251 Da) or rather bulky (536–694 Da) aromatic substituents attached to the C5 position of the pyrimidine ring of the dUTPs localized in the active center of the enzyme.

Amino acid residues Arg587, Arg659, Arg660, Ala661, and Thr664 belong to the active center of the enzyme [32,36,37,38,39,40,41] while Glu530 and Phe647 extend beyond the active center facing cavity A while Val586, Arg595, Asp610, Pro656, Leu657, Glu820, and Lys831 extend beyond the active center facing cavity B. The non-covalent interactions with 4 of the 14 amino acid residues (Arg587, Arg660, Ala661, and Thr664) were detected experimentally and published previously [36,37,38,39,40,41]. The ten amino acid residues (Glu530, Val586, Arg595, Asp610, Phe647, Pro656, Leu657, Arg659, Glu820, and Lys831) are first predicted in this paper as capable of forming non-covalent bonds with dNTP substituents. The abovementioned amino acid residues can be used as a target in directed mutagenesis to create new polymerases with desirable specificity to the modified dNTP substituents.

## 4. Materials and Methods

### 4.1. dUTPs Modified at C5-Position with Bulky or Small Aromatic Substituents

The chemical structures of dUTPs C5-modified with bulky aromatic substituents (536–694 Da, including linker -CH=CH-CH_2_-NHCO-(CH_2_)_5_- and bulky aromatic groups, which are Cy5+, Cy5±, or Cy5–) or with lighter hydrocarbon substituents R0–R7 (126–251 Da, including linker -CH=CH-CH_2_-NHCO-CH_2_- and -CH_2_-CH_3_ or small aromatic groups) are shown in Figure 1. The synthesis and spectroscopic characteristics of the corresponding modified dUTPs (dU(Cy5±)TP, dU(Cy5+)TP, and dU(Cy5–)TP) as well as PCR efficiency of dUTPs modified with lighter low molecular weight substituents R0–R7 were described earlier [28,47].

### 4.2. The DNA Template and Primers

The 68-nucleotide DNA template and 18- and 17-nucleotide-long primers labeled with Cy3 dye were the same as those used in [28]:

Template:

5′-TCTCTTGCCCTTTCGTC*T*C*T*AAA*TT*G*T*C*TT*AA*T*C*T*C*TT*C*T*A*T*CC*TT*C*T*C*T*C*T*CACCAC*TT*ACA*T*CCGC-3′

Primer P1-Cy3:

Cy3-NH-5′-GCGGATGTAAGTGGTGAG-3′

Primer P2-Cy3:

Cy3-NH-5′-TCTCTTGCCCTTTCGTC-3′

Primer sequences and the corresponding primer binding sites are underlined. Adenines subject to complementary binding with modified deoxyuridines of the enzymatically synthesized DNA strand are underlined by wavy lines. Thymines that can be replaced with modified deoxyuridines during PCR are shown in italics. The embedding of modified nucleotides complementary to the template is significantly difficult in sites containing clusters of complementary bases. Therefore, the sequence of the DNA template specifically included both the isolated A and isolated AA and AAA repeats to study the ability of the polymerase to use modified dUTPs complementary to these A-repeats. In addition, the template sequence was chosen to avoid the formation of self-complementary structures as much as possible. 

### 4.3. PCR and Electrophoresis

PCR was performed following a protocol similar to the one described previously [28]. DNA polymerases of different organisms were used, namely:

Thermus aquaticus (Taq);
Thermus thermophilus (Tth);
Pyrococcus furiosus (Pfu) DNA polymerases (Sileks, Badenweiler, Germany) as well as
Thermococcus litoralis (Vent);
Pyrococcus GB-D strain (Deep Vent) DNA polymerases and forms of the native DNA polymerases;
Thermococcus litoralis (Vent (exo-)); and
Pyrococcus GB-D strain (Deep Vent (exo-)) that had been genetically engineered to eliminate the 3′→5′ proofreading exonuclease activity (New England Biolabs, Ipswich, UK).

Each polymetrase’s PCR conditions were selected for efficient amplification with natural dNTPs. Herein, the amount of PCR products amplified with the polymerases was the same for all the considered polymerases within an accuracy of 25%, which allowed for facile observation, registration, and comparison of the electrophoregrams.

The PCR conditions for each of the polymerases and the detailed protocols for PCR amplification, gel electrophoresis, and obtaining gel images are presented in “Part G: PCR and electrophoresis” in the supplementary data.

### 4.4. Quantitative Analysis of Electrophoretic Bands Containing PCR-Amplified Full-Length DNA Fragments

The relative quantities of the PCR-amplified full-length DNA fragments containing modified dUMPs were estimated by the relative fluorescence intensity of the Cy3 dye conjugated to oligonucleotide primers (λ^abs^_max_ = 551 nm, λ^em^_max_ = 567 nm [47]). These quantities were calculated separately for each of the elecrophoregram lanes. For this purpose, the summed Cy3 fluorescence intensity of the bands containing full-length DNA fragments with incorporated modified dUMPs was quantified using a virtual rectangular frame that surrounded the bands. The fluorescence intensities of all the pixels surrounded by the frame were summed to obtain the total fluorescence intensity within the frame. The total fluorescence intensity of the blank gel region within the same frame of the same size was then subtracted from the obtained value. 

To obtain the relative values of PCR efficiency, the fluorescence intensity of the DNA products containing incorporated modified dUMPs was normalized to the product obtained in the presence of only natural dNTPs.

The relative fluorescence intensity of the Cy5 labeled dUMPs incorporated into DNA strands (λ^abs^_max_ = 648 nm, λ^em^_max_ = 670 nm [53]) also characterized the relative quantity of the PCR-amplified full-length DNA fragments. Cy5 fluorescence intensity of the bands containing full-length DNA product in each lane was summarized with the same approach, which was used to calculate PCR efficiency in the Cy3 fluorescence range. The obtained quantities were normalized to the maximum value.

### 4.5. Computer Modeling of 3D Structures of Triple Complexes Consisting of KlenTaq Polymerase–DNA–(Modified dUTP)

3D molecular modeling was carried out using the Small Molecules and Receptor–Ligand Interactions modes found in the Discovery Studio program [49] and based on known X-ray structures [33,40,48] to localize the substituents at the C5 position of the pyrimidine ring of dUTPs in the active center of the KlenTaq polymerase.

3D structures of the complexes (1QTM and 5E41) determined by X-ray crystallography [33,40,48] were rigidly fixed. The structures of the corresponding deoxypyrimidine triphosphates localized in the active center were also rigidly fixed in accordance with the covalent docking procedure. Water molecules were deleted. Structural solutions were obtained as a result of optimizing the energy of the conformation of the substituents (linker–functional groups, see Figure 1) and the energy of its interactions with polymerase amino acid residues and DNA.

The types and quantities of the detected non-covalent bonds between the substituent and polymerase were compared with the experimental data regarding the efficiency of PCR amplification in the presence of the corresponding modified dUTPs in the PCR mixture to find possible correlations.

### 4.6. KlenTaq Polymerase Amino Acid Residues’ Ability to Form Non-Covalent Interactions with the dUTP Substituents Attached at the C5 Position of the Pyrimidine Ring Localized in the Active Center of the Enzyme

The capability of KlenTaq polymerase amino acid residues to non-covalently interact with the substituents attached at the C5 position of the pyrimidine ring of dUTP localized in the active center of the enzyme was estimated quantitatively. The mean number of non-covalent bonds formed by a particular amino acid residue per one structural solution, n, was calculated as:

n = N/S,

where N was the number of non-covalent bonds formed between a particular amino acid residue and the substituents by summing over all the structural solutions. S was the total number of structural solutions obtained in modeling.

## 5. Conclusions

In this work, the following structural–functional relationships were estimated or proposed for the substituents of modified dNTPs:

(i) The PCR efficiency in the presence of dUTPs modified with low molecular weight substituents (126–694 Da) decreases with an increase in the number of non-covalent bonds between the substituents and the DNA polymerase (about 15% decrease per one extra non-covalent bond). The decrease in the efficiency of PCR can be explained by the necessity of spending extra energy to overcome these additional bonds in order to release the polymerase molecule for further moving along the DNA primer/template after the formation of a phosphodiester bond;
(ii) The number of non-covalent bonds between the dNTP substituents and polymerase amino acid residues can be considered as a variable parameter for regulation of enzyme activity. This number of non-covalent bonds can be estimated by modeling with the help of the highly efficient Discovery Studio software and can be useful in future design and synthesis of dNTP derivatives with new polymerase-specific properties;
(iii) The obtained results demonstrate the similarity of all the tested DNA polymerases of the A and B families in the ability to use a substrate that mimics dTTP—the dUTP modified with a certain substituent. One can assume that, despite the varieties in the amino acid chains forming the active centers of different polymerases, the chemical groups of the amino acid residues facing the spaces of the active centers as well as those facing the adjacent regions (possibly forming cavities similar to the cavities A and B of the KlenTaq polymerase) form similar local environments not only for dNTPs (which is well known), but also for their substituents.
(iv) The ten amino acid residues of Taq polymerase (Glu530, Val586, Arg595, Asp610, Phe647, Pro656, Leu657, Arg659, Glu820, and Lys831) are first predicted as capable of forming non-covalent bonds with dNTP substituents. The abovementioned amino acid residues can be considered the useful targets in directed mutagenesis to create mutant polymerases with desirable specificity to the modified dNTP substituents.

## Supplementary Materials

The supporting information can be downloaded at: https://www.mdpi.com/article/10.3390/ijms241713643/s1.
