# Supplementary material for: Non-Covalent Interactions between dUTP C5-Substituents and DNA Polymerase Decrease PCR Efficiency
Source: Int J Mol Sci. 2023 Sep 4;24(17):13643. doi: 10.3390/ijms241713643 (PMC10487964; doi:10.3390/ijms241713643)
Supplement: Supplementary file 1 [file ijms-24-13643-s001.zip › Supplementary_pdf_+_22_3D_figures__dsv/Supplementary_information_to_correlations.pdf]

Supplementary information

Non-covalent interactions between dUTP C5-substituents and DNA polymerase decrease PCR efficiency

Olga A. Zasedateleva, Sergey A. Surzhikov, Viktoriya E. Kuznetsova, Valeriy E. Shershov, Victor E. Barsky, Alexander S. Zasedatelev, Alexander V. Chudinov

Engelhardt Institute of Molecular Biology, Russian Academy of Sciences, 32 Vavilov Street, 119991 Moscow, Russia

Contents

Part A: Inhibiting effect of modified dUTPs in the reaction mixture on the yield of PCR product consisting only of natural nucleotides.

Part B: Table S1.

Part C: Interactions between the small dUTP substituents (functional groups R0 - R7 attached via a linker -CH=CH-CH_2-NHCO-CH_2- to the C5 position of pyrimidine ring) and amino acid residues of KlenTaq polymerase. Table S2.

Part D: Interactions between the bulky dUTP substituents (differently charged Cy5 dye analogs attached via a linker -CH=CH-CH_2-NHCO-$(CH_2)_5$- to the C5 position of pyrimidine ring) and amino acid residues of KlenTaq polymerase. Figures S1-S3 and Table S3.

Part E: The similarity of local environments in the active centers of various polymerases to the dUTP substituents attached at the C5 position of the pyrimidine ring.

Part F: Table S4.

Part G: PCR and electrophoresis.

Part H: References.

Part A: Inhibiting effect of modified dUTPs in the reaction mixture on the yield of PCR product consisting only of natural nucleotides.

As can be seen from Figure 2A of the main text, full-size PCR products synthesized using each of the tested polymerases in the presence of only natural dNTPs form two closely spaced electrophoretic fluorescent bands corresponding to the elongation products of the direct primer (upper band) and the reverse primer (lower band) (lanes 2, 6. 10, 15, 19, 23 and 27). The calculated difference in the masses of these DNA strands is 1012 Da.

These closely spaced doublet bands characterizing the amounts of PCR-products consisted of only natural nucleotides almost completely disappear for the cases of amplification by any of the studied polymerases, when 5% dTTP in reaction mixtures are replaced with dU(Cy5+)TP (lanes 3, 7, 11, 16, 20, 24 and 28) or with dU(Cy5±)TP (lanes 4, 8, 12, 17, 21, 25 and 29). At the same time, when 5% of dTTP are replaced with dU(Cy5-)TP (lanes 5, 9, 13, 18, 22, 26 and 30), the intensity of the doublet bands of the DNA product, consisting only of natural nucleotides, practically does not decreases.

It can be concluded that even low concentrations ($5x10^{-6}$ M) of dU(Cy5+)TP or dU(Cy5±)TP in the content of the conventional PCR mixture used in this study lead to a significant inhibition of incorporation of natural nucleotides by polymerase. However, the similar inhibition of polymerase activity does not occur when dU(Cy5-)TP is contained in reaction mixture at the same concentration.

The inhibiting effect similar to the one described above was observed earlier when studying the effect of these modified dUTPs on reaction of primer elongation by Taq polymerase along a template that even did not contain adenines, i.e. did not contain complementary bases for incorporation dU(Cy5+)MP, dU(Cy5±)MP or dU(Cy5-)MP [1].

Part B: Table S1.

Table S1. The efficiency of PCR amplification by A family (Taq and Tth) and B family (Pfu, Vent (exo-), Deep Vent (exo-), Vent and Deep Vent) DNA polymerases in the presence of 5% dUTPs modified via linker -$CH=CH-CH_2-NHCO-(CH_2)_5$- at the C5 position of pyrimidine rings with bulky aromatic substituents (536-694 Da) containing the analogs of Cy5 dye - zwitterionic neutrally charged Cy5±, positively charged, Cy5+, or negatively charged Cy5-, as compared to that when only natural dNTPs were used. The data summarize the results presented in Figure 2 of the main text.

Detection range	Polymerases / Abbeviations for dUTPs labeled with Cy5 dye analogs	Family A			Family B					
		Taq	Tth	Averaged values of PCR efficiency	Pfu	Vent (exo-)	Deep Vent (exo-)	Vent	Deep Vent	Averaged values of PCR efficiency
Cy3 range	dU(Cy5±)TP	0,435± 0.119	0,618± 0.201	0,526± 0.092	0,523± 0.126	0,267± 0.064	0,150± 0.008	0,133± 0.014	0,109± 0.045	0.236± 0.127
	dU(Cy5+)TP	0,058± 0,026	0,061± 0,011	0,059± 0.002	0,228± 0,009	0,170± 0,028	0,046± 0,004	0,057± 0,006	0,022± 0,002	0,105± 0,076
	dU(Cy5-)TP	0,038± 0,012	0,073± 0,021	0,055± 0.018	0,101± 0,006	0,067± 0,025	0,069± 0,004	0,048± 0,008	0,041± 0,031	0.065± 0.017

Part C: Interactions between the small dUTP substituents (functional groups R0 - R7 attached via a linker -CH=CH-CH_2-NHCO-CH_2- to C5 position of pyrimidine ring) and amino acid residues of KlenTaq polymerase. Table S2.

Table S2. Number of hydrogen, carbon hydrogen, π-donor hydrogen, electrostatic π-cation and π-anion and hydrophobic π-alkyl bonds between the substituents (functional groups R0 - R7 attached via a -CH=CH-CH_2-NHCO-CH_2- linker) and amino acid residues of KlenTaq polymerase. The data were obtained using molecular modeling of these substituents in KlenTaq polymerase–DNA–(dUTP or ddTTP) 3D complex, (PDB IDs: 5E41 and 1QTM, [2-4]). Functional groups are listed in descending order of relative yield of PCR products amplified by Taq polymerase when replacing dTTP with dUTPs modified with these substituents. Relative amounts of PCR products averaged over Taq, Tth, Vent (exo-) and Deep Vent (exo-) polymerases are also shown.

Functional group, R, attached via linker	Relative PCR yield using Taq polymerase [5]	Relative PCR yield averaged over polymerases [5]	Number of hydrogen bonds		Number of carbon hydrogen bonds		Number of π-donor hydrogen bonds		Number of electrostatic π - cation bonds		Number of electrostatic π - anion bonds		Number of hydrophobic π - alkyl bonds		Mean number of non-covalent bonds
			5E41	1QTM	5E41	1QTM	5E41	5E41	5E41	1QTM	5E41	1QTM	5E41	1QTM	
-	1	1	-	-	-	-	-	-	-	-	-	-	-	-	-
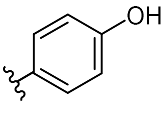 (R1)	0.81±0.04	0.76±0.13	1 (Arg660)	1 (Arg660)	0	0	0	0	1 (Arg587)	0	0	0	2 (Arg660, Ala661)	1 (Arg660)	3±1.0
-CH_2-CH_3 (R0)	0.57±0.07	0.70±0.07	3 (Thr664, Arg660(2))	1 (Arg660)	0	0	0	0	0	0	0	0	0	0	2.0±1.0
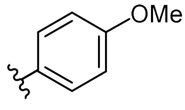 (R3)	0.55±.03	0.47±0.08	2 (Arg660)	1 (Arg660)	0	0	1 (Thr664)	0	1 (Arg587)	1 (Lys831)	0	1 (Asp610)	0	0	3.5±0.5
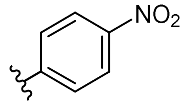 (R5)	0.52±.02	0.38±0.14	1 (Arg660)	1 (Arg660)	0	0	1 (Thr664)	0	1 (Arg587)	0	0	1 (Asp610)	0	0	2.5±0.5
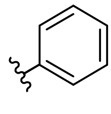 (R4)	0.51±0.02	0.42±0.15	1 (Arg660)	1 (Arg660)	0	0	1 (Thr664)	0	1(Arg587)	0	0	0	0	1 (Arg660)	2.5±0.5
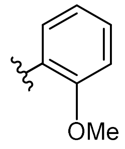 (R2)	0.39±0.01	0.33±0.16	1 (Arg660)	1 (Arg660)	0	2 (Val586, Arg660)	0	0	1 (Arg587)	0	0	0	3 (Arg587, Arg660, Ala661)	1 (Arg660)	4.5±0.5
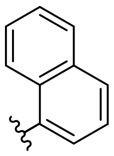 (R6)	0.18±0.03	0.09±0.09	1 (Thr664)	1 (Arg660)	1 (Arg660)	0	0	0	2 (Arg587)	1 (Lys831)	0	0	5 (Arg587(2), Arg660 (2), Ala661)	1 (Lys831)	6.0±3.0
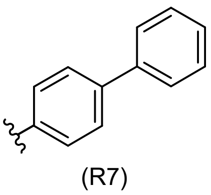 (R7)	0.09±0.01	0.05±0.04	3 (Arg660 (2), Thr664)	1 (Arg660)	0	0	0	0	1 (Arg587)	2 (Arg659, Lys831)	0	0	1 (Ala661)	0	4.0±1.0

Part D: Interactions between the bulky dUTP substituents (differently charged Cy5 dye analogs attached via a linker -CH=CH-CH_2-NHCO-$(CH_2)_5$- to the C5 position of pyrimidine ring) and amino acid residues of KlenTaq polymerase. Figures S1-S3 and Table S3.

Figure S1.

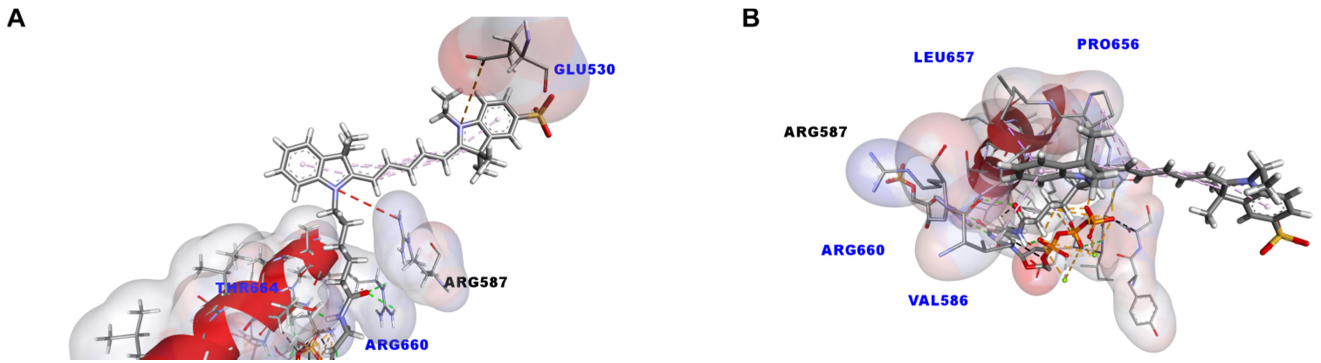

Figure S1. Non-covalent interactions of a substituent of dU(Cy5±)TP with amino acid residues of KlenTaq polymerase obtained using molecular modeling and covalent docking.
A. The substituent introduced into the structure 5E41 formed 4 non-covalent bonds:
1. - 2. two hydrogen bonds between oxygen atom of the NHCO linker group and NH and NH_2 groups of Arg660,
3. hydrogen bond between the NH linker group and oxygen atom of Thr664,
4. attractive charge bond between nitrogen atom of far from linker indolyl group and oxygen atom of Glu530,
There is an unfavorable positive-positive interaction (red dotted line) between the nitrogen atom of close to the linker indolyl ring and the nitrogen atom of Arg587.
B. The substituent introduced into the structure 1QTM formed 6 non-covalent bonds:
1. hydrogen bond between oxygen atom of NHCO group of linker and nitrogen atom of Arg660,
2. - 4. three alkyl bonds between carbon atoms of middle hydrocarbon chain as well as of close to linker indolyl group of fluorophore group and pentagonal ring of Pro656,
5. π-sigma bond between aromatic ring of close to the linker indolyl group and hydrogen atom of Leu657,
6. π-alkyl bond between aromatic ring of close to linker indolyl group and methyl group of Val586,
See also 3D visualization for the results of docking using the Discovery Studio program [6] (supplementary Figure4C_Cy5+-.dsv and Figure4D_Cy5+-.dsv files).

Figure S2.

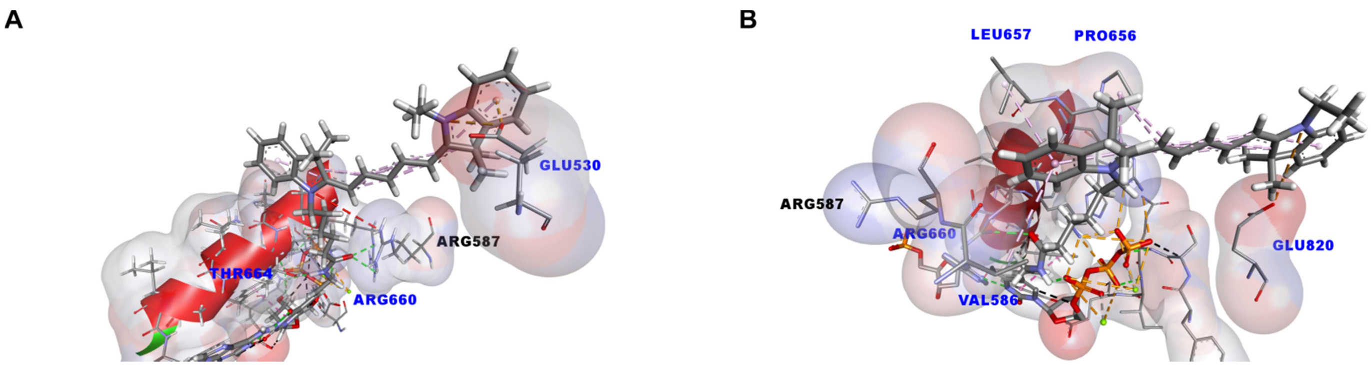

Figure S2. Non-covalent interactions of a substituent of dU(Cy5+)TP with amino acid residues of KlenTaq polymerase obtained using molecular modeling and covalent docking.
A. The substituent introduced into the structure 5E41 formed 5 non-covalent bonds:
1. - 2. two hydrogen bonds between oxygen atom of the NHCO linker group and NH and NH_2 groups of Arg660,
3. hydrogen bond between the NH linker group and oxygen atom of Thr664,
4. attractive charge bond between nitrogen atom of far from linker indolyl group and oxygen atom of Glu530,
5. π-anion bond between aromatic ring of far from linker indolyl group and oxygen atom of Glu530,
There is an unfavorable positive-positive interaction (red dotted line) between the nitrogen atom of close to the linker indolyl ring and the nitrogen atom of Arg587.
B. The substituent introduced into the structure 1QTM formed 7 non-covalent bonds:
1. hydrogen bond between oxygen atom of the NHCO group of linker and nitrogen atom of Arg660,
2. attractive charge bond between nitrogen atom of far from linker indolyl group and oxygen atom of Glu820,
3. - 5. three alkyl bonds between carbon atoms of middle hydrocarbon chain as well as of close to linker indolyl group of fluorophore group and pentagonal ring of Pro656,
6. π-alkyl bond between aromatic ring of close to the linker indolyl group and methyl group of Leu657,
7. π-alkyl bond between aromatic ring of close to linker indolyl group and methyl group of Val586.
See also 3D visualization for the results of docking using the Discovery Studio program [6] (supplementary Figure4C_Cy5+.dsv and Figure4D_Cy5+.dsv files).

Figure S3.

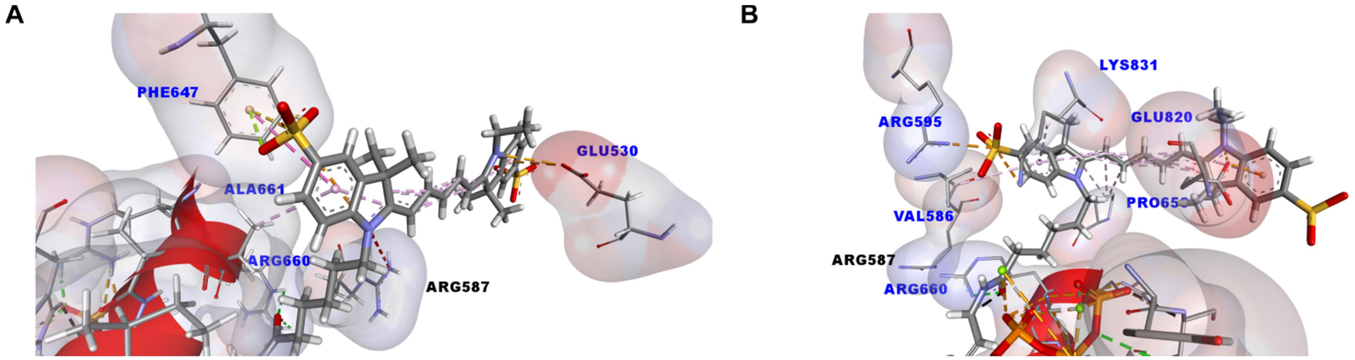

Figure S3. Non-covalent interactions of a substituent of dU(Cy5-)TP with amino acid residues of KlenTaq polymerase obtained using molecular modeling and covalent docking.

A. The substituent introduced into the structure 5E41 formed 8 non-covalent bonds:

1. - 2. two hydrogen bonds between oxygen atom of the NHCO linker group and NH and NH_2 groups of Arg660,
3. attractive charge bond between nitrogen atom of far from linker indolyl group and oxygen atom of Glu530,
4. π-anion bond between aromatic ring of far from linker indolyl group and oxygen atom of Glu530,
5. π-alkyl bond between aromatic ring of close to linker indolyl group and carbon atom of Ala661,
6. π-π stacked bond between aromatic ring of close to linker indolyl group and that of Phe647,
7. π-sulfur bond between sulfur atom of close to linker SO_3 group and aromatic ring of Phe647,
8. π-lone pair bond between oxygen atom of close to linker SO_3 group and aromatic ring of Phe647.

There is an unfavorable positive-positive interaction (red dotted line) between the nitrogen atom of close to the linker indolyl ring and the nitrogen atom of Arg587.

B. The substituent introduced into the structure 1QTM formed 10 non-covalent bonds:

1. hydrogen bond between oxygen atom of NHCO group of linker and nitrogen atom of Arg660,
2. attractive charge bond between nitrogen atom of far from linker indolyl group and oxygen atom of Glu820,
3. π-anion bond between aromatic ring of far from linker indolyl group and oxygen atom of Glu820,
4. π-alkyl bond between aromatic ring of close to linker indolyl group and methyl group of Val586,
5. π-alkyl bond between aromatic ring close to linker indolyl group and hydrocarbon chain of Lys831,
6. - 8. three alkyl bonds between carbon atoms of middle hydrocarbon chain as well as of close to linker indolyl group of fluorophore group and pentagonal ring of Pro656,
9. attractive charge bond between SO_3 group which is close to linker and nitrogen atom of Lys831,
10. attractive charge bond between SO_3 group which is close to linker and nitrogen atom of Arg595.

See also 3D visualization for the results of docking using the Discovery Studio program [6] (supplementary Figure4C_Cy5-.dsv and Figure4D_Cy5-.dsv files).

Table S3. Number of hydrogen, electrostatic (attractive charge and π-anion bonds), π-lone pair, π-sulfur bonds and hydrophobic bonds (π-π stacked, π-sigma, alkyl and π-alkyl bonds) between the bulky substituents (differently charged Cy5 dye analogs attached via -CH=CH-CH_2-NHCO-$(CH_2)_5$- linker) and amino acid residues of KlenTaq polymerase. The data were obtained using molecular modeling of these substituents in KlenTaq polymerase–DNA–(dUTP or ddTTP) 3D complex, (PDB IDs: 5E41 and 1QTM, [2-4]). Functional groups are listed in descending order of relative yield of PCR products amplified by Taq polymerase when partial (5%) replacing dTTP with dUTPs modified with these substituents. Relative amounts of PCR products averaged over Taq, Tth, Pfu, Vent (exo-), Deep Vent (exo-), Vent and Deep Vent polymerases are also shown.

Functional group, R, attached via linker	Relative PCR yield using Taq polymerase	Relative PCR yield averaged over polymerases	Number of hydrogene bonds		Number of electrostatic attractive charge bonds		Number of π-sulfur bonds		Number of electrostatic π-anion bonds		Number of π – lone pair bonds		Number of hydrophobic π – π stacked bonds		Number of hydrophobic π-sigma bonds		Number of hydrophobic alkyl bonds		Number of hydrophobic π - alkyl bonds		Mean number of non-covalent bonds
			5E41	1QTM	5E41	1QTM	5E41	1QTM	5E41	1QTM	5E41	1QTM	5E41	1QTM	5E41	1QTM	5E41	1QTM	5E41	1QTM	
-	1	1	-	-	-	-	-	-	-	-	-	-	-	-	-	-	-	-	-	-	-
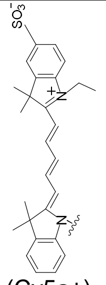 (Cy5a±)	0.44±0.12	0.32±0,18	3 (Arg660 (2), Thr664)	1 (Arg660)	1 (Glu530)	0	0	0	0	0	0	0	0	0	0	1 (Leu657)	0	3 (Pro656)	0	1 (Val586)	5.0±1.0
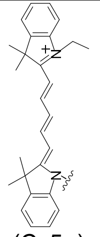 (Cy5+)	0.06±0.03	0.09±0.06	3 (Arg660 (2), Thr664)	1 (Arg660)	1 (Glu530)	1 (Glu820)	0	0	1 (Glu530)	0	0	0	0	0	0	0	0	3 (Pro656)	0	2 (Leu657, Val586)	6.0±1.0
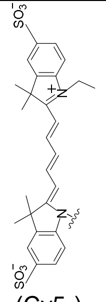 (Cy5-)	0.04±0.01	0.06±0.02	2 (Arg660)	1(Arg660)	1 (Glu530)	3 (Arg595, Glu820, Lys831)	1 (Phe647)	0	1 (Glu530)	1 (Glu820)	1 (Phe647)	0	1 (Phe647)	0	0	0	0	3 (Pro656)	1 (Ala661)	2 (Val586, Lys831)	9±1.0

Part E: The similarity of local environments in the active centers of various polymerases to the dUTP substituents attached at the C5 position of the pyrimidine ring.

As was already mentioned in the paragraph 3.2.5 of the main text, there is considerable similarity between two types of dependences of the relative efficiency of PCR-amplification in the presence of modified dUTPs on the mean number of non-covalent bonds between dUTP substituent and polymerase. The first type of dependences was obtained for the case when the relative PCR-efficiency of Taq polymerase was plotted against the mean number of non-covalent interactions between substituent and Taq polymerase (see Figures 5A and C of the main text). The second type of dependences was obtained for the case when the relative PCR-efficiency averaged over all tested polymerases was plotted along the ordinate axis against the same values of the mean number of non-covalent bonds between substituent and Taq polymerase (see Figures 5B and D of the main text). The described similarity between the first and the second types of dependences is clearly observed as in the case of relatively light (126-251 Da) low molecular weight dUTP substituents (as follows from comparison of Figures 5A and B) and in the case of rather bulky substituents (536 - 694 Da) (see Figures 5C and D of the main text for comparison).

The noted similarity leads one to conclude that despite the varieties in the sequences of amino acid chains forming the active centers of different polymerases, the atomic groups of amino acids facing the spaces of active centers, as well as facing adjacent regions (possibly forming cavities similar to cavities A and B of KlenTaq polymerase), form similar local environments in relation not only to dUTP, but also to its substituents attached at the C5 position of the pyrimidine ring. This circumstance can contribute to the formation of similar quantity of non-covalent substituent-polymerase bonds, which leads to a noticeable analogy in the plots shown in Figures 5A and B and also in Figures 5C and D of the main text.

Part F: Table S4.

Table S4. Summarized number of bonds, N, and averaged number of bonds, n, between amino acid residue of KlenTaq polymerase and substituent at the C5 position of pyrimidine ring of dUTP obtained using molecular modeling. Averaging was performed by normalizing N per the number, S, of structural solvings obtained in modeling: n = N/S (see Materials and Methods, Paragraph 2.6.)					
Lighter low molecular weight substituent (carrying R0-R7 group, S=16 structural solvings)			Bulky aromatic low molecular weight substituent (S=6 structural solvings)		
A.a.r.	N	n	A.a.r.	N	n
Val586	1	0,0625	Glu530	5	0,833333
Arg587	11	0,6875	Val586	3	0,5
Asp610	2	0,125	Arg595	1	0,166667
Arg659	1	0,0625	Phe647	3	0,5
Arg660	27	1,6875	Pro656	9	1,5
Ala661	4	0,25	Leu657	2	0,333333
Thr664	6	0,375	Arg660	9	1,5
Lys831	4	0,25	Ala661	1	0,166667
			Thr664	2	0,333333
			Glu820	3	0,5
			Lys831	2	0,333333

Part G: PCR and electrophoresis.

The PCR conditions for the polymerases were as follows. All reaction mixtures (25 µl) for PCR amplification of the 68-nucleotide template contained each primer (10^{-6} M), the template ($4x10^{-9}$ M), each deoxynucleoside triphosphate (dATP, dCTP, dGTP, dTTP, 10^{-4} M, or $0.95x10^{-4}$M dTTP and $0.05x10^{-4}$M modified dUTP (at 5% addition of modified dUTP)), and the manufacturer's recommended buffer (70 mM Tris HCl, 16.6 mM $(NH_4)_2SO_4$, 2 mM $MgCl_2$, pH 8.6, for Taq, Tth and Pfu polymerases, and 20 mM Tris HCl, 10 mM $(NH_4)_2SO_4$, 10 mM KCl, 2 mM $MgSO_4$, 0.1% Triton® X-100, pH 8.8, for Vent, Deep Vent, Vent (exo-) and Deep Vent (exo-) polymerases). In the case of PCR amplification by Vent (exo-) polymerase, the reaction mixture additionally contained 8% formamide.

Tubes containing the reaction mixture without polymerase were placed into the heating block of a Peltier thermal cycler (Dyad from Bio-Rad, Hercules, CA, USA) and heated at 95 °C for 6 min. After that, the temperature was decreased to 62 °C, which is the calculated melting temperature of the P1-Cy3 and P2-Cy3 primers. Immediately after heating for 6 min at 62 °C, the appropriate amounts of polymerase (3 units of Taq polymerase, 1 unit of Tth polymerase, 15 units of Pfu, 0.12 units of Vent polymerase, 0.12 units of Deep Vent polymerase, 0.5 units of Vent (exo-) polymerase or 0.08 units of Deep Vent (exo-) polymerase) were added to the reaction mixtures, which were then stirred without removing the tubes from the block. This excluded the possibility that the activity of the polymerases decreased during the pre-denaturation stage. Then, 20 reaction cycles of heating at 94 °C for 1 min and cooling to 62 °C for 1.5 min were conducted.

The reactions were terminated with 450 µl of 2.5% (for the case of amplification using Taq and Tth polymerases) or 5% (for the case of amplification using Pfu, Vent, Deep Vent, Vent (exo-) and Deep Vent (exo-) polymerases) lithium perchlorate trihydrate in 95% ethanol to form 90% of the final ethanol concentration followed by short-term mixing and 18 hours of precipitation at -20 °C. After that the samples were centrifuged for 10 min at 13000 rpm using Biofuge pico centrifuge (Kendro Laboratory Products GmbH, Hanau, Germany) and the supernatants were removed. Then the samples were washed with 40 µl of 95% ethanol with lithium perchlorate trihydrate at the same corresponding concentrations at 20 °C for 5 min and 450 rpm using Thermomixer comfort (Eppendorf, Hamburg, Germany), exposed for centrifugation for 4 min at 13000 rpm using Biofuge pico centrifuge. The supernatants were removed.

Then the samples were dried at 37 °C for 10 min using Termo 24-15 thermostat (Biocom, Moscow, Russia). Each sample was dissolved in 6µl of water solution of 7M Urea and 0.01% Bromophenol blue and heated at 95 °C for 50 s before being loaded into gel wells.

The PCR products were separated by electrophoresis in 18% polyacrylamide denaturing gels ((19:1 (w/w) acrylamide/bis-acrylamide, 7 M urea; 700 V; thermostabilized 16 × 16 cm glass sandwich with 1-mm gel thickness; TBE buffer (89 mM Tris-borate and 2 mM ethylenediaminetetraacetic acid, pH 8.3)) using PROTEAN II xi cell (Bio-Rad Laboratories Inc., Hercules, CA, USA) and an Elf-8 power supply (DNA Technology, Moscow, Russia).

After electrophoresis, images of the gels were obtained in the Cy3 and Cy5 fluorescence ranges (λ^{abs}_{max}=550 nm, λ^{em}_{max}=570 nm, or λ^{abs}_{max}=648 nm, λ^{em}_{max}=670 nm, respectively, as described in the manufacturer’s instructions for Amersham CyDye mono-reactive NHS Esters) using a gel imager for fluorescence spectroscopy with an image field of 20×16 cm, as described earlier [1,5]. The gel imager was equipped with an RTE/CCD-1536-K/1 CCD camera (Roper Scientific, Sarasota, FL, USA), a mercury lamp, a 535DF35 and 580DF27 filter pair and a 630DF30 and 690DF40 filter pair (Omega Optical, Brattleboro, VT, USA) and a computer running ImaGel Research software [1,5].

Part H: References.

1. Zasedateleva, O.A.; Vasiliskov, V.A.; Surzhikov, S.A.; Kuznetsova, V.E.; Shershov, V.E.; Guseinov, T.O.; Smirnov, I.P.; Yurasov, R.A.; Spitsyn, M.A.; Chudinov, A.V. dUTPs conjugated with zwitterionic Cy3 or Cy5 fluorophore analogues are effective substrates for DNA amplification and labelling by Taq polymerase. *Nucleic Acids Res.* **2018**, *46*, e73.

2. Li, Y.; Mitaxov, V.; Waksman, G. Structure-based design of *Taq* DNA polymerases with improved properties of dideoxynucleotide incorporation. *Proc. Natl. Acad. Sci. USA.* **1999**, *96*, 9491–9496.

3. Hottin, A.; Betz, K.; Diederichs, K.; Marx, A. Structural Basis for the KlenTaq DNA Polymerase Catalysed Incorporation of Alkene- versus Alkyne-Modified Nucleotides. 2017. Chemistry, 23 (9), 2109-2118. https://doi.org/: 10.1002/chem.201604515

4. https://www.rcsb.org Berman H.M., Westbrook J., Feng Z., Gilliland G., Bhat T.N., Weissig H., Shindyalov I.N. and Bourne P.E. The Protein Data Bank. *Nucleic Acids Res.* **2000**, *28*, 235-242. (accessed on 31 July 2023)

5. Zasedateleva, O.A.; Surzhikov, S.A.; Shershov, V.E.; Miftakhov, R.A.; Yurasov, D.A.; Kuznetsova, V.E.; Chudinov, A.V. PCR incorporation of dUMPs modified with aromatic hydrocarbon substituents of different hydrophilicities: Synthesis of C5-modified dUTPs and PCR studies using Taq, Tth, Vent (exo-) and Deep Vent (exo-) polymerases. *Bioorg Chem.* **2020**, *99*, 103829.

6. BIOVIA, Dassault Systèmes, Discovery Studio, 2020, San Diego: Dassault Systèmes, 2020. https://discover.3ds.com/discovery-studio-visualizer-download (accessed on 31 July 2023)
